# A comparative study of α-Ni(OH)_2_ and Ni nanoparticle supported ZIF-8@reduced graphene oxide-derived nitrogen doped carbon for electrocatalytic ethanol oxidation†

**DOI:** 10.1039/d3ra08208c

**Published:** 2024-02-13

**Authors:** Soliman Gamal, Doaa A. Kospa, Amr Awad Ibrahim, Awad I. Ahmed, A. M. A. Ouf

**Affiliations:** a Chemistry Department, Faculty of Science, Mansoura University Al-Mansoura 35516 Egypt amr_awad@mans.edu.eg

## Abstract

Ethanol electrooxidation is an important reaction for fuel cells, however, the major obstacle to ethanol electrocatalysis is the splitting of the carbon–carbon bond to CO_2_ at lower overpotentials. Herein, a ZIF-8@graphene oxide-derived highly porous nitrogen-doped carbonaceous platform containing zinc oxide was attained for supporting a non-precious Ni-based catalyst. The support was doped with the disordered α-phase Ni(OH)_2_ NPs and Ni NPs that are converted to Ni(OH)_2_ through potential cycling in alkaline media. The Ni-based catalysts exhibit high electroactivity owing to the formation of the NiOOH species which has more unpaired d electrons that can bond with the adsorbed species. From CV curves, the EOR onset potential of the α-Ni(OH)_2_/ZNC@rGO electrode is strongly shifted to negative potential (*E*_onset_ = 0.34 V) with a high current density of 8.3 mA cm^−2^ relative to Ni/ZNC@rGO. The high catalytic activity is related to the large interlayer spacing of α-Ni(OH)_2_ which facilitates the ion-solvent intercalation. Besides, the porous structure of the NC and the high conductivity of rGO facilitate the kinetic transport of the reactants and electrons. Finally, the catalyst displays a high stability of 92% after 900 cycles relative to the Ni/ZNC@rGO and commercial Pt/C catalysts. Hence, the fabricated α-Ni(OH)_2_/ZNC@rGO catalyst could be regarded as a potential catalyst for direct EOR in fuel cells.

## Abstract 2

Ethanol electrooxidation is an important reaction for fuel cells, however, the major obstacle to ethanol electrocatalysis is the splitting of the carbon–carbon bond to CO_2_ at lower overpotentials.

## Graphical abstract

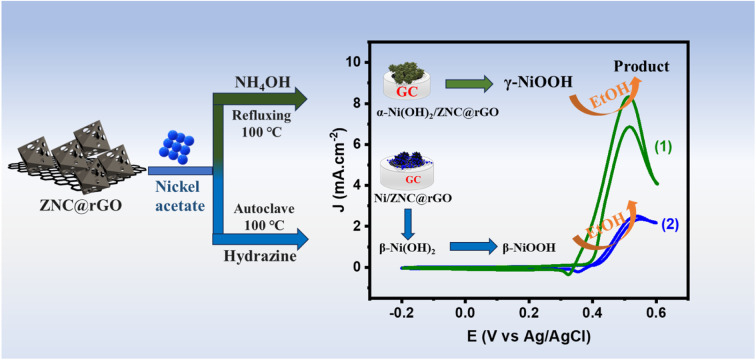

## 1. Introduction

Even now, petroleum derivatives meet the majority of global energy demand.^1^ Thus, the production of alternative technologies for energy storage is critical for future generations. To date, researchers are searching for alternative renewable energy resources that are clean, low-cost, and commercially available.^2,3^ Fuel cell devices attract widespread attention instead of rechargeable commercial batteries due to providing portable energy sources, poisoning resistance, low-cost fuel, and clean byproducts.^2^ Because of their extraordinarily high specific energy, alcoholic fuel cells are favorable electronic devices compared to modern batteries. However, the wide utilization of methanol (direct methanol fuel cells, DMFCs) is limited by its toxicity unlike ethanol (direct ethanol fuel cells, DEFCs), which is less toxic. On the other hand, enormous amounts of ethanol can be manufactured from biomasses or agricultural products.^4^ The ideal mechanism of complete oxidation entails the splitting of the carbon–carbon (C–C) bond and multiple dehydrogenations that involve a 12-electron pathway CH_3_CH_2_OH + 12OH^−^ → 2CO_2_ + 9H_2_O + 12e^−^.^5^ The major obstacle to ethanol electrocatalysis is the high overpotentials required for the C–C splitting to CO_2_.^6,7^

Generally, the DEFC performance is determined by the electrocatalyst activity in the ethanol oxidation reaction (EOR).^8^ The more negative onset potential which describes the potential in an electrochemical cell that drives the forward reaction, indicates the high catalytic activity for EOR. Recently noble metal-based electrocatalysts including Pd or Pt and their alloys with transition elements are the most efficient catalysts for EOR.^9–11^ However, the poisoning of electrocatalytic active sites of noble metals by CO, scarcity, and high costs which affect the stability of electrocatalysts limit their potential utilization. Therefore, researchers focus their efforts on developing easily available, cost-effective, and more stable electrocatalysts for fuel cells.^12^ Non-precious transition metals (Zn, Fe, Cu, Ni, and Co) and their oxides, sulfides, and hydroxides are promising electrocatalysts with remarkable activity and durability for EOR.^8,12,13^ Ni is one such element that stands out among non-noble metals for a variety of energy-related applications.^14^ Nickel-based electrocatalysts are remarkable materials for various applications including hydrogen evolution, supercapacitor, water splitting, CO_2_ reduction, and oxygen reduction.^15,16^ Ni-based active sites promote the oxidation of the electroactive species *via* peroxide formation which enhances the conductivity, increases the active site number and develops the electron transfer from the modified electrode to the electrolyte decreasing the reaction overpotential and facilitates catalytic activity.^17–23^ Moreover, nickel hydroxides (Ni(OH)_2_) have attracted great attention in electrochemical applications including supercapacitors, Ni-based alkaline batteries, and electrochemical sensors owing to their high stability under hard conditions.^24^ The Ni(OH)_2_-based catalysts exhibit high electrocatalytic activity because they are oxidized to highly active nickel oxyhydroxide (NiOOH) species which can bond with the adsorbed species through empty or unpaired d-orbitals.^17^ Despite that Ni(OH)_2_ normally has two polymorphs (alpha and beta), the alpha polymorph (α-Ni(OH)_2_) is preferred for electrochemical applications owing to several factors including; (i) the α-phase is oxidized through the electrochemical reactions to γ-NiOOH and both have more disordered structures which facilitate the ion-solvent intercalation due to the large interlayer spacing,^25^ (ii) β-Ni(OH)_2_ is oxidized to β-NiOOH with lower oxidation state (+3) compared to that of γ-NiOOH (+3.5) resulting in lower oxidation potentials.^24^ Moreover, the α-Ni(OH)_2_-based catalysts are electrochemically preferred to Ni NPs which are converted to the more thermodynamically favorable ordered structure (β-phase Ni(OH)_2_) under the applied potential in alkaline media.^26^

However, due to their insufficient conductivity and low surface area, it is necessary to combine electron donors including carbon-based nanomaterials (carbon nanotubes and graphenes) and/or transition metal oxides (TMOs) to overcome these limits.^27^ According to recent studies, carbon networks with heteroatom coordination and TMOs have been used to improve the electrocatalytic oxidation of ethanol.^28^ Meanwhile, the introduction of metal oxide/carbon-based materials facilitates electron transport improving the electric conductivity.^29^ In comparison to the traditional metal oxide/nitrogen-doped carbon catalysts with disorder structures, some newly hierarchical carbonaceous structures were investigated for superior electrochemical performance.^28^ Metal–organic frameworks (MOFs) are suitable precursors for the synthesis of carbonaceous platforms containing metal oxide nanocomposites with various topologies.^30^ MOFs are porous structure materials with high crystallinity that have been created with several morphologies and tunable characteristics for different applications.^31–34^ MOFs are distinguished by high porosity and surface area, the difference in pore size, structure, shape, high conductivity, and catalytic activity.^32,35–38^ Through the pristine MOFs, the active sites for redox reactions were enclosed by the organic ligands inhibiting their conductivity.^39^ However, the high-temperature thermal treatment can produce highly conductive porous materials as the organic linker is transformed into a porous carbonaceous platform and metal cations are oxidized to metal oxide which are uniformly dispersed on the carbon surface.^40^

Among all MOFs, ZIFs called zeolitic imidazolate framework (also called zeolitic metal azolate framework) exhibit exclusive, distinct, and highly desirable properties like crystallinity, high surface area, microporosity, in addition to excellent chemical and thermal stability because it has both zeolites and metal–organic framework.^41^ ZIF-8 which consists of (Zn^2+^) as a metal cation linked to methylimidazole as a linker, is the most studied ZIF as it possesses a highly porous structure.^42,43^ ZIF-8 has a high conductivity due to nitrogen from imidazolate which plays a role in various applications like sensors, gas storage, catalysis, and electrocatalytic energy production.^44^ In addition to its unique features, it should be noted that the pyrolysis of ZIF-8 generates nitrogen-doped carbon, which enhances the electron transfer rate, and the interaction with the electrolyte and reactants because the nitrogen functional groups possess n-type or metallic behaviour.^45^ Besides, nitrogen doping promotes the high stability of the active species toward ethanol oxidation.^46^ Moreover, the derived ZnO nanoparticles from ZIF can exhibit favourable properties for EOR such as non-toxicity, high catalytic efficiency, high surface area, chemical stability and strong adsorption ability.^47^

Recently, 2D materials have attracted great attention in the scientific community for electronic, optoelectronic, and various energy applications owing to their superior electrical, mechanical, and optical properties compared to their layered bulk counterparts.^48–52^ The pioneer of these materials is graphene which has sparked global interest in various applications such as energy storage, biosensors, electrochemical sensors, wearable devices, and the textile industry owing to its exceptional properties.^53–58^ GO possesses a large surface area and selectivity in alcohol adsorption from water which facilitates the ethanol oxidation process. In another aspect, graphene oxide (GO) has attracted great attention as conducting support owing to its improved chemical stability and higher electrical conductivity and surface area.^59,60^ The electrical conductivity is due to the e-conjugation along the structural sp^2^ carbon centre and the associated anchoring sites which make GO good support for uniform dispersion of small particles.^59,61^ However, the strong oxidants used through the process of GO synthesis introduce various defects in its crystal structure, resulting in its low conductivity in comparison to graphene.^53^ To solve this issue, GO can be reduced and converted into reduced graphene oxide (rGO) which possesses similar features to graphene.

Herein, for the first time, the α-Ni(OH)_2_/ZNC@rGO combination was reported as an electrocatalyst for efficient electrochemical ethanol oxidation at lower overpotential. Zinc oxide/nitrogen-doped carbon deposited on the rGO surface (ZNC@rGO) was derived from the ZIF-8@rGO thermal decomposition under the N_2_ atmosphere at 700 °C. Subsequently, the alfa nickel hydroxide was precipitated on the surface through the refluxing of nickel acetate-impregnated ZNC@rGO in an alkaline medium. For comparison, the Ni/ZNC@rGO which is converted to more ordered β-phase Ni(OH)_2_ in alkaline media during the electrochemical tests, was synthesized by the hydrothermal method. To ensure that the electrocatalysts were successfully manufactured, several characterizations were performed. Finally, the fabricated electrocatalysts were tested by several electrochemical techniques to study their electrochemical activities for ethanol oxidation. The α-Ni(OH)_2_/ZNC@rGO electrocatalyst exhibited an efficient activity for the EOR at a more negative onset potential (0.34 V *versus* Ag/AgCl) with 8.3 mA cm^2^ current density. These results are due to the carbon-based material and ZnO nanoparticles which promote the OH^−^ adsorption at low potentials facilitating the active site formation (γ-NiOOH). While the Ni/ZNC@rGO electrocatalyst exhibited 80 mV more positive than that for oxidation of α-Ni(OH)_2_/ZNC@rGO electrocatalyst owing to the slow transformation of β-Ni(OH)_2_/β-NiOOH species. Additionally, the catalyst displays a high stability of 92% after 900 cycles compared to Ni/ZNC@rGO and the commercial Pt/C catalysts.

## 2. Materials and method

### 2.1. Materials and reagents

All purchased chemicals were utilized without any additional purification. *N*,*N*′-Dimethyl formamide (DMF, ASC), zinc nitrate (Zn(NO_3_)_2_·6H_2_O), 2-methyl imidazole, Pt/C (20%), and sulfuric acid (H_2_SO_4_) from Sigma-Aldrich. Nafion (5%), nickel(ii) acetate (Ni(OAc)_2_·4H_2_O), hydrochloric acid (HCl), absolute ethanol (C_2_H_5_OH, 99.99%), methanol (CH_3_OH), and hydrogen peroxide (H_2_O_2_) were obtained from Fischer Scientific. Potassium permanganate (KMnO_4_), sodium nitrate (NaNO_3_), and graphite powder (99.9998%) were obtained from Sigma-Aldrich.

### 2.2. Synthesis of electrocatalysts

#### 2.2.1. Synthesis of pristine ZIF-8

As reported in the previous literature, ZIF-8 was synthesized as follows;^62^ initially, Zn(NO_3_)_2_·6H_2_O (2.5 mmol) was mixed with 2-methyl imidazole (5 mmol) in 40 ml methanol and stirred for complete dissolving to form a clear solution. After that, the mixture was kept in a Teflon autoclave at 120 °C for 10 hours. The white precipitate was cooled to room temperature, centrifuged with a rate of 4000 rpm, and washed several times with DMF and ethanol before overnight drying at 70 °C yielding 0.6 g of ZIF-8.^63,64^

#### 2.2.2. Synthesis of graphene oxide support ZIF-8 (10% rGO/ZIF-8)

Firstly, graphene oxide (GO) was obtained from the oxidation of natural graphite powder based on the modified Hummers' approach.^65^ Graphite powder (5.0 g) was typically dispersed in 115 ml of H_2_SO_4_ (96%) with 29.4 mmol of NaNO_3_ (2.5 g) under powerful stirring in an ice bath for 1 h. Subsequently, 189.8 mmol of KMnO_4_ (30 g) was steadily mixed with the reaction till the formation of a brown slurry. After the complete addition of KMnO_4_, the slurry was removed from the ice bath and completed under powerful stirring for a half-hour at 40 °C. Then, 350 ml of hot water was dropwisely added to the reaction under stirring, and then 650 ml of cold water was added. After that, 15 ml of H_2_O_2_ (30%) was added to the reaction till the complete change of the brown colour to pale yellow. Finally, the obtained precipitate was collected and washed five times with 1 l of DI water with 1% HCl followed by washing four times before drying at 60 °C.^59^

Next, the as-synthesized graphene oxide was utilized for further preparation of 10% rGO/ZIF-8. In 40 ml methanol, 0.06 g of GO was dispersed under vigorous sonication with a frequency of 40 kHz for 30 min and then added to 2.5 mmol of Zn(NO_3_)_2_·6H_2_O which was stirred till the complete dissolution of zinc nitrate. After 15 min, 40 ml methanol containing 10 mmol of 2-methyl imidazole was slowly added to the previous reaction and kept for 10 h at 120 °C after transferring into an autoclave. Finally, the composite was collected for centrifugal washing with DMF and ethanol before overnight drying at 70 °C.

#### 2.2.3. Synthesis of zinc oxide/nitrogen-doped carbon@reduced graphene oxide (ZNC@rGO)

The rGO/ZIF-8 was carbonized under an N_2_ atmosphere in a furnace adjusted at 700 °C for 2 hours with a 5 °C min^−1^ ramp rate. The final catalyst was collected, washed twice with ethanol, dried at 60 °C, labelled as ZNC@rGO, and stored for the next steps.

#### 2.2.4. Synthesis of 10% Ni/ZNC@rGO

1.0 g of the ZNC@rGO support was ultrasonicated in 10 ml DI water for 1 h till the complete suspension in water. A solution of nickel acetate (1.7 mmol ∼0.42 g in 10 ml DI water) was added to the previous suspension under stirring for 1 hour followed by the mixing of hydrazine hydrate (10 ml of 1 M). After that, the solution was kept in a furnace in a Teflon autoclave for 22 hours at 100 °C. The final composite was cooled to room temperature, centrifuged, washed with DI water and dried at 70 °C for 24 h.

#### 2.2.5. Synthesis of 10% α-Ni(OH)_2_/ZNC@rGO

1.0 g of the ZNC@rGO support was suspended in 60 ml ethanol for 1 h and then 1.1 mmol of nickel(ii) acetate (0.27 g) was dissolved under vigorous stirring. After that, 5 ml NH_4_OH was mixed with the previous solution and then refluxed for 3 h at 100 °C until the precipitation of the pale green nickel hydroxide on the surface. The resulting precipitate Ni(OH)_2_/ZNC@rGO was washed twice with DI water and centrifuged before drying at 70 °C for 24 h.

### 2.3. Materials characterization

To characterize the as-prepared materials, many analysis approaches were applied. A Nicolet iS10 FT-IR spectrophotometer with a diamond attenuated total reflectance (DATR) was used to verify the functional groups' existence. Thermal gravimetric analysis (TGA) was performed to investigate the thermal characteristics of the materials. A certain amount of each catalyst was enclosed in an aluminium pan and heated to 800 °C at a heating rate of (10 °C min^−1^). An X-ray powder diffraction instrument (XRD; Bruker device) was used to investigate the bulk crystallinity of the fabricated catalysts which was applied at a 2-theta angle ranging between 10 and 70° with monochromatic radiation (Cu-K_α_, *λ* = 1.54 Å). The N_2_ adsorption–desorption test was performed using a NOVAtouch 4LX instrument at the pressure of 1 bar and 77 K after the degassing of each at 180 °C overnight. After that, the adsorption data in a relative pressure range was used to calculate the Brunauer–Emmett–Teller surface area. A transmission electron microscopy (TEM) utilizing a Joel JEM-2100 apparatus was applied to investigate the sample's morphologies. Moreover, the morphological structures of the samples were tested by scanning electron microscopy (SEM) using JEOL JSM 6510 lv with EDX techniques, which were mounted on the SEM stub at a 90° angle and coated with gold for 30 seconds in a sputter chamber. For surface elements identification and quantification, the Thermo Fisher Scientific (ESCALAB 250) apparatus was used to record the sample's X-ray photoelectron spectroscopy (XPS). The electrochemical measurements were applied to all prepared catalysts with a three-configuration system with a Versastat 3500 potentiostat.

### 2.4. Electrochemical tests

#### 2.4.1. Preparation of the working electrodes

A glassy carbon electrode (GC, 3 mm diameter) was polished on a smooth emery paper with aqueous alumina suspensions and washed with DI water followed by acetone till it became like a mirror. For the working electrode preparation, each catalyst (2 mg) was suspended in 1 ml ethanol containing 10 μl of Nafion (5%) at room temperature till a slurry formed. Subsequently, 10 μl of this slurry was homogenously dispersed onto the cleaned GC electrode till the formation of a thin layer, dried, and used for further electrochemical tests.

#### 2.4.2. Electrochemical measurements

A conventional three-configuration system was utilized to perform electrochemical experiments in 1 M KOH at room temperature. The system consists of the modified glassy carbon (working electrode), Ag/AgCl, KCl sat (reference electrode), and a platinum wire (counter electrode). The calculated electrochemical surface area was used to normalize the achieved current density. All potentials in this study were recorded under an N_2_-saturated solution and referred to the reference Ag/AgCl. In the potential window ranging between −0.2 and 0.6 V, the cyclic voltammetry (CV) technique was applied at a scan rate of 50 mV s^−1^. Moreover, the CV measurements were performed from −0.1 to 0.0 V at various scan rates of 20–150 mV s^−1^ to evaluate the electrochemical active surface area (ECSA) of the electrocatalysts which was conducted as follows:^35,36^1
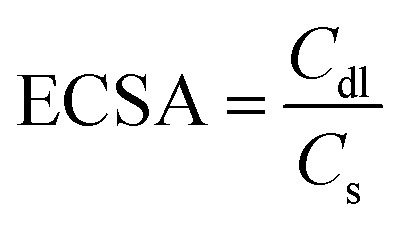
where *C*_s_ and *C*_dl_ are the specific capacitance of the catalysts (0.035 mF cm^−2^) in an alkaline medium for nickel-based catalysts and the capacitance of the electrochemical double layer which is counted using the slope of the linear fitting of the current density *versus* scan rates, respectively.

Besides, the CV curves were studied for the different catalysts in the range of −0.2 to 0.6 V at different scan rates (20–300 mV s^−1^) in the presence of 1 M ethanol in 1 M KOH. The diffusion coefficient (*D*, in m^2^ s^−1^) can be calculated from the linear relation between the peak current density (*I*_p_, in amperes) *versus* the square root of the scan rate (*ν*^1/2^) based on the Randlese–Sevcik equation as follow:^66,67^

2 *I*_p_ = 2.69 × 10^5^*n*^3/2^*AD*^1/2^*v*^1/2^*C*

where *n*, *C* and *A* represent the electron number in the rate-determining step, the ethanol concentration (in mole per cm^3^), and the electrode surface area, respectively.

Meanwhile, the linear relationship between the scan rates and the anodic/cathodic peaks can be used to calculate the surface coverage (τ*) according to the following equation:^68,69^3
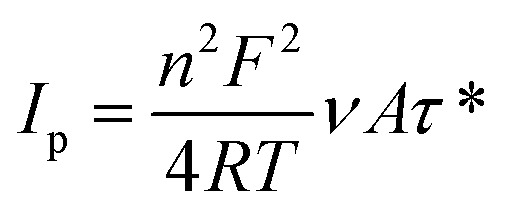


As *I*_p_ represents the peak current density, *n* is the electrons number, *T* = 298 K is the room temperature, *ν* is the potential scan rate (mV s^−1^), *R* = 8.314 J K^−1^ mol^−1^ is the ideal gas constant, *A* is the geometric surface area of the GC electrode, and *F* is the Faraday constant (96 485 C mol^−1^).

At a scan rate of 50 mV s^−1^ from −0.2 to 0.6 V, the CV technique was performed in the presence of different ethanol concentrations (0.1–2 M) in 1 M KOH. Besides, the linear sweep voltammetry (LSV) was recorded at 10 mV s^−1^. Moreover, the electrochemical impedance spectroscopy (EIS) under a potential of 0.6 V was investigated at different temperatures in 1 M KOH + 1 M EtOH solution in the range of 0.01 Hz to 10^5^ Hz with an amplitude of 10 mV. The CV measurement was performed for 900 cycles to indicate the stability and durability of the fabricated catalysts. At 0.5 V, the chronoamperometry (CA) technique was applied for 3000 s in a solution of 1 M ethanol and 1 M KOH, and from the Cottrell law, the diffusion coefficient (*D*) was determined:^2^4
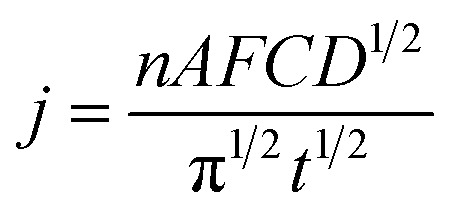
where *j*, *A*, *n*, *C*, *F*, and *t* are the current density (mA), the GC electrode surface area (cm^2^), the number of electrons, the concentration of ethanol (M), the Faraday constant, and the time of EOR (s), respectively.

## 3. Result and discussion

### 3.1. Physicochemical characterization

**Fig. 1:**
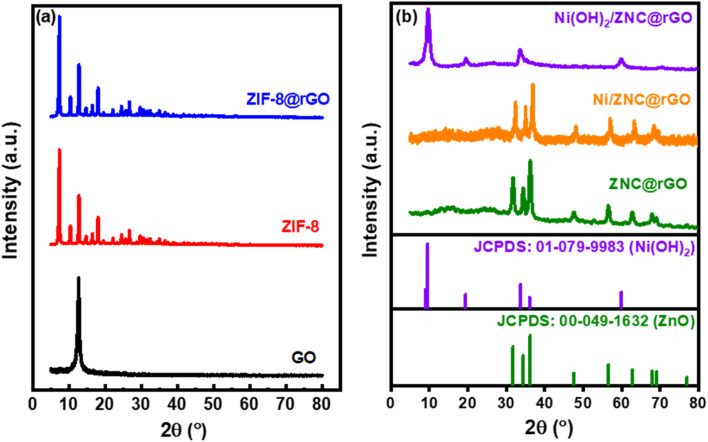
XRD pattern of (a) GO, ZIF-8, and ZIF@rGO and (b) ZNC@rGO, Ni/ZNC@rGO, and Ni(OH)_2_/ZNC@rGO.

The crystallinity of the fabricated catalysts was determined using the XRD technique and the recorded data are represented in Fig. 1a and b. For ZIF-8 (Fig. 1a), the strong peaks are observed at 2*θ* = 7.2 (110), 10.4 (200), 12.7 (211), 14.6 (220), 16.5 (310), 18.0 (222), 24.5 (233), and 26.6° (134) indicating the high crystallinity of the as-synthesized ZIF-8 particles.^64^ The characteristic peak of GO was observed at 2*θ* of 12.6° which is reduced after the combination with ZIF-8 in ZIF-8@rGO structure (Fig. 1a). The pattern of the ZNC@rGO (Fig. 1b) shows various peaks at 2*θ* of 31.6 (100), 34.5 (0 0 2), 36.2 (101), 47.7 (102), 56.5 (110), 62.6 (103), and 68.2° (112) indicating that the derived ZnO nanoparticles exhibit the wurtzite structure (JCPDS no. 00-049-1632).^64,70^ Moreover, the XRD pattern of Ni/ZNC@rGO (Fig. 1b) shows identical diffraction peaks of the pure ZNC@rGO with high crystallinity and a small shift to higher two theta value due to the incorporation of the Ni nanoparticles on the ZnO lattice.^71^ The pattern of Ni(OH)_2_/ZNC@rGO (Fig. 1b) displays the characteristic peaks of the α-Ni(OH)_2_ nanoparticles at 9.9, 19.5, 33.6, 39.0, 52.7, and 60.0° attributing to the (001), (100), (110), (111), (103) and (201) reflections (JCPDS no. 01-079-9983), respectively.^72^ Some of the characteristic peaks of ZNC and rGO are not as clear as those of pure ZNC@rGO due to the sharp peaks of alpha nickel hydroxide.

The corresponding N_2_ adsorption–desorption isotherms and pore distributions of ZIF-8, ZIF-8@rGO, ZNC@rGO, and Ni(OH)_2_/ZNC@rGO are displayed in Fig. S1 (ESI).† The adsorption–desorption isotherm (Fig. S1a†) of ZIF-8 shows a typical type (I) Langmuir isotherm (IUPAC classification) indicating the microporous structure of ZIF-8.^73^ The sample exhibits a large BET surface area of 911 m^2^ g^−1^ with a total pore volume of 0.35 cm^3^ g^−1^. Compared to the pure ZIF-8, the specific surface area of the ZIF-8@rGO catalyst decreases from 911 m^2^ g^−1^ to 536 m^2^ g^−1^ with a slight decrease in the pore volume to 0.24 cm^3^ g^−1^ confirming the occupation of a certain amount of the pores in the ZIF-8 structure by rGO. The pore size distribution for both materials is in the range of 0.5–1.5 nm (Fig. S1b†). After the pyrolysis of ZIF-8@rGO, the surface area of the derived ZNC@rGO decreases to 41 m^2^ g^−1^ with a decrease of the pore volume reaching up to 0.015 cm^3^ g^−1^ confirming the strong scaffold of the derived ZnO/carbon matrix. The distributions of the pore diameter are mostly centred at 2–6 nm indicating the mesoporous structure of the derived sample. For the Ni(OH)_2_/ZNC@rGO, the curve type can be regarded as type (IV) indicating the presence of mesopores (Fig. S1a†). The surface area was found to be 27 m^2^ g^−1^ with a pore volume of 0.001 cm^3^ g^−1^ and a maximum peak centred at approximately 4.3 nm which would be beneficial for the electron diffusion (Fig. S1b†).

**Fig. 2:**
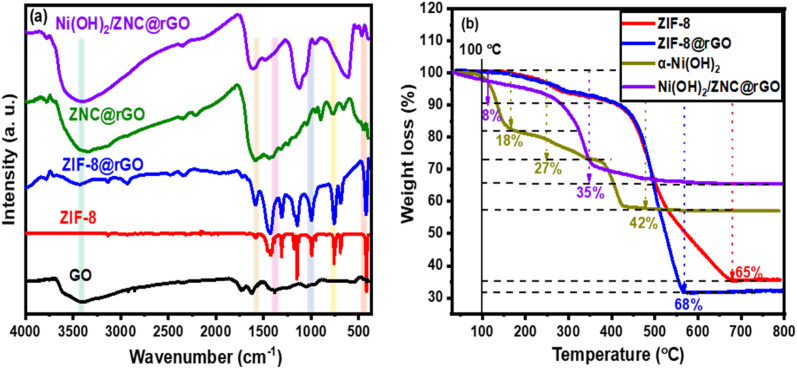
(a) FT-IR spectra of all prepared catalysts and (b) TGA analysis of ZIF-8, ZIF-8@rGO, α-Ni(OH)_2_, and α-Ni(OH)_2_/ZNC@rGO.

To further confirm the successful fabrication of the proposed catalysts, the FT-IR technique was used as represented in Fig. 2a. The characteristic peaks of ZIF-8 are observed at 3138 and 2986 cm^−1^ belonging to the stretching of the C–H bond in C=C and the methyl group (–CH_3_), respectively.^64,74^ Additionally, the band at 1578 cm^−1^ is related to the C=N stretching through the ring of imidazole while the peaks from 1400 to 1100 cm^−1^ are corresponding to the C–N stretching. Moreover, the bands at 994 and 420 cm^−1^ are associated with the swing of the N–H bond and the N–Zn stretching, respectively. The spectrum of the GO shows various oxygen configurations in the structure including the hydroxyl groups (C–OH) from COOH and H_2_O at 3650–3150 cm^−1^.^75,76^ Besides, the characteristic peaks at 2919.8, 1734, 1401, 1220, and 957 cm^−1^ are corresponding to the C–H, ketonic species (C=O), the O–H deformation, stretching vibration of the C–O group, and peroxide group, respectively.^77,78^ ZIF@rGO spectrum showed both the characteristic peaks of GO and ZIF-8 with very low intensities of the peaks at 3650–3150 cm^−1^ related to the complete GO reduction to rGO.^75^ Compared to the peaks of ZIF-8, the disappearance of the Zn–N stretch band (420 cm^−1^) in the ZNC@rGO spectrum with a new observed peak at 459 cm^−1^ due to the Zn–O vibration confirmed the pyrolysis of the pristine MOF.^70,79^ For the Ni(OH)_2_/ZNC@rGO spectrum, there are three sharp peaks at 3390, 1615, and 626 cm^−1^ corresponding to the O–H stretching in Ni(OH)_2_ and H_2_O, the bending mode of O–H group in the interlayer H_2_O, and the stretching mode of Ni–O–H bond, respectively.^80^

The thermal stability of ZIF-8, ZIF-8@rGO, α-Ni(OH)_2_, and Ni(OH)_2_/ZNC@rGO catalysts were tested by the TGA analysis and the diagram is shown in Fig. 2b. It is observed that weight loss of ZIF-8 and ZIF-8@rGO materials consisted of three steps. The first stage is owing to the loss of adsorbed water on the catalyst surface which occurred from room temperature to 160 °C with a mass loss of 5.2%. Moreover, the removal of guest molecules (mainly H_2_O) from the cavities of the ZIF-8 structure begins in the second stage from 160 to 380 °C with a mass loss of approximately 10%. The final step is attributed to the decomposition of GO and the removal of the organic linker molecules of ZIF-8 which occurred over the temperature range from 380 to 670 °C and 660 °C with a total mass loss of 65% and 74% for ZIF-8 and ZIF-8@rGO, respectively. It is noted that the weight loss of the pure ZIF-8 sample in the final stage is constant at 65%, while the ZIF-8@rGO presents an obvious weight loss (9%) due to the combustion of the rGO. Hence, the mass percentage of the graphene oxide in the composite is 9%. On the other hand, the TGA plot of the pure α-Ni(OH)_2_ exhibits four weight loss regions over the temperature range. The first weight loss occurred below 110 °C with a value of 2% which is due to the removal of adsorbed water molecules. The second stage of 11.6% weight loss from 110 to 200 °C is owing to the elimination of interlayer water molecules. The third weight loss of 27% from 200 to 380 °C is attributed to the decomposition of Ni(OH)_2_ to NiO. Finally, the fourth stage with a weight loss of 42% from 380 to 440 °C is due to the elimination of interlayer anions (CO_3_^2−^ and NO_3_^−^). For the Ni(OH)_2_/ZNC@rGO, the decomposition occurred in two stages; the first stage is with a weight loss of 8% below 200 °C due to the removal of the adsorbed water. While the second stage in the range from 200 to 550 °C exhibits a total weight loss of 35% due to the decomposition of Ni(OH)_2_ to NiO.

**Fig. 3:**
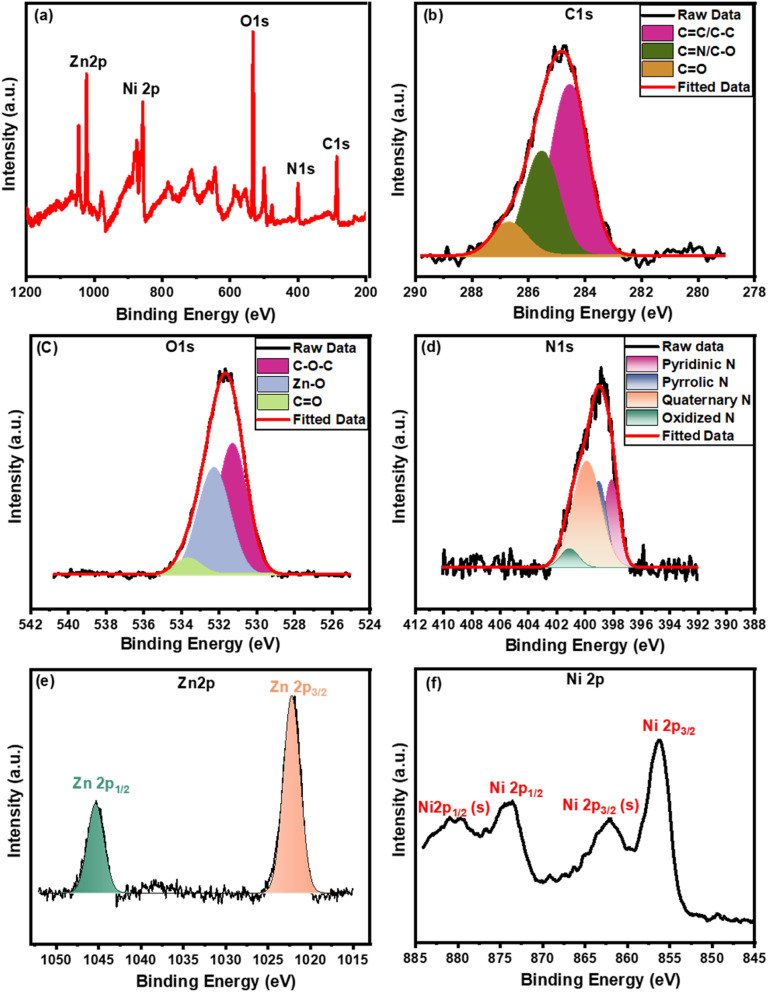
XPS analysis of Ni(OH)_2_/ZNC@rGO (a) total survey and (b–f) the corresponding high-resolution spectrum of C 1s, O 1s, N 1s, Zn 2p, and Ni 2p, respectively.

The elemental composition and its oxidation states can be quantitatively confirmed using the XPS measurements. The total survey spectrum of the GO (Fig. S2a†) designates the sample to contain C and O elements without any other impurities. The high-resolution spectrum of the C 1s (Fig. S2b†) exhibits four Gaussian peaks at 284.2, 285.4, 286.9, and 288.5 eV which belong to the C–C/C=C, C–OH/C=C, C–O–C, and HO–C=O, respectively.^60,81^ Meanwhile, the spectrum of O 1s in Fig. S2c† is fitted with three peaks related to the C=O (531.2 eV), C–O–C/C–OH (533.3 eV), and adsorbed water on the surface (535.9 eV). On the other hand, Fig. 3a exhibits the Ni(OH)_2_/ZNC@rGO total survey spectrum which contains C, O, N, Zn, and Ni elements. The C 1s spectrum (Fig. 3b) is fitted to three peaks at 286.7, 285.5, and 284.5 eV related to the C=O, C=N/C–O, and C–C/C=C bonds, respectively.^82,83^ It was observed that the characteristic peak of the COOH group disappeared and the intensities of oxygen-functional groups reduced relative to GO (Fig. S2†) indicating the successful reduction of the graphene oxide to reduced graphene oxide in the composite.^72,84^Fig. 3c represents the XPS spectrum of O 1s which displays three Gaussian signals at 531.1 eV (C–O–C), 532.3 eV (Zn–O), and 533.7 eV (C=O).^85^ Moreover, the N 1s spectrum is displayed in Fig. 3d exhibiting four fitted peaks at 398.1, 399.2, 399.9, and 401.1 eV related to the different functionalized nitrogens including the pyridinic N, pyrrolic N, quaternary N, and oxidized N, respectively, indicating that N atoms embedded in carbon network.^86,87^ For Zn 2p, the XPS spectrum is deconvoluted into two peaks at 1045.1 eV (Zn 2p_1/2_) and 1021.9 eV (Zn 2p_3/2_) with a distance of 23.2 eV indicating the presence of Zn^2+^ (Fig. 3e).^85^ The XPS Ni 2p spectrum in Fig. 3f shows two major peaks of Ni 2p_1/2_ (873.7 eV) and Ni 2p_3/2_ (856.2 eV) associated with two satellites at 880.3 eV and 862.1 eV, respectively. The energy separation between the two major signals is found to be 17.5 eV which confirms the formation Ni^2+^ oxidation state of alpha nickel oxide.^72,88^

**Fig. 4:**
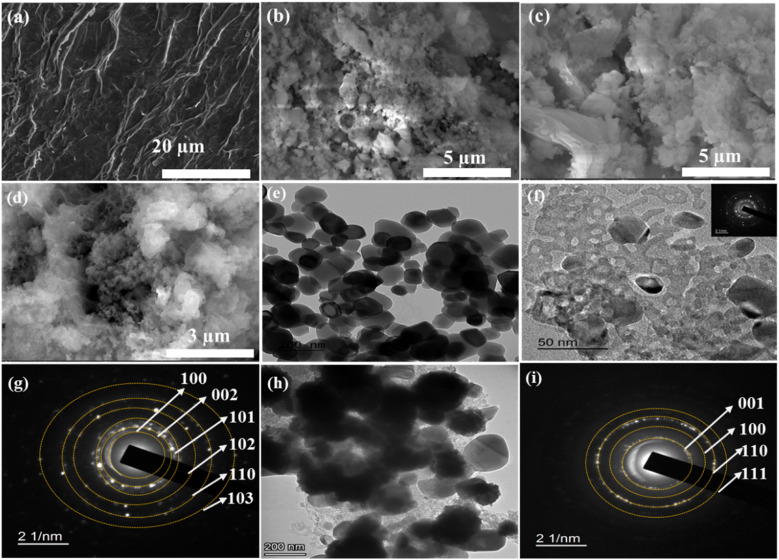
SEM images of (a) GO, (b) ZNC, (c) ZNC@rGO, and (d) α-Ni(OH)_2_/ZNC@rGO, and TEM images of (e) ZIF-8, (f) ZNC@rGO, and (h) Ni(OH)_2_/ZNC@rGO, and the corresponding SAED patterns of (g) ZNC@rGO and (i) Ni(OH)_2_/ZNC@rGO.

Moreover, SEM and TEM analyses were performed to understand the morphological structures of the fabricated catalysts (Fig. 4). The SEM image of rGO in Fig. 4a shows a two-dimensional sheet-like structure. Besides, the image displays the numerous lamellar layers that characterize the GO structure.^89^ SEM images of ZNC (Fig. 4b) and ZNC@rGO (Fig. 4c) show the distribution of highly cohesive ZnO nanoclusters on the surface of the carbon support derived from ZIF-8 and ZIF-8@rGO, respectively.^90^ The conducted SEM images of Ni(OH)_2_/ZNC@rGO are displayed in Fig. 4d and S3a† which show the growth of a thin layered spongy flake-like porous structure on the surface of the ZNC@rGO.^88^ However, owing to the embedded alpha nickel hydroxide structure, the morphology of ZNC@rGO is not clearly visible in the SEM images, even at higher magnifications. Thus, HRSEM elemental mapping and EDX analysis were performed to validate the presence of the elemental composition of the Ni(OH)_2_/ZNC@rGO layers. The elemental mapping region of the catalyst is displayed in Fig. S3b† which confirms the growth of a thin layered spongy flake-like porous structure on the surface. The overlay mapping image is shown in Fig. S3c† confirming that the Ni(OH)_2_ nanoparticles are preferentially dispersed on the support surface. The elemental mappings confirm that C, O, N, Zn, and Ni are dispersed evenly across the whole mapping region as shown in Fig. S3(d–h),† respectively. The results exhibit strong evidence for the Ni(OH)_2_ existence on the ZNC@rGO surface. Besides, the elemental composition of the Ni(OH)_2_/ZNC@rGO electrode was quantified using EDX analysis. The presence of C, N, O, Zn, and Ni elements in Fig. S4† demonstrates the effective synthesis of Ni(OH)_2_/ZNC@rGO.

The TEM analysis was carried out for the fabricated materials and the images are represented in Fig. 4(e–i). The TEM image of ZIF-8 particles (Fig. 4e) exhibits the rhombic dodecahedron shape with truncated corners.^91^ Additionally, Fig. 4f shows the TEM image of ZNC@rGO which exhibits a good distribution of the small nanoparticles of the ZnO polyhedron on the highly porous carbon matrix-wrapped graphene oxide sheets.^64,92^ From the TEM image of Ni(OH)_2_/ZNC@rGO (Fig. 4h), the Ni(OH)_2_ nanospheres are uniformly dispersed on the ZNC@rGO surface with a small particle size of 20–60 nm. Moreover, the corresponding selected area electron diffraction (SAED) pattern of ZNC@rGO and Ni(OH)_2_/ZNC@rGO was conducted. The SAED pattern of ZNC@rGO (Fig. 4g) consists of six concentric diffraction rings corresponding to (001), (100), (110), (111), (103) and (201) reflections. On the other hand, the bright spots in the SAED pattern of Ni(OH)_2_/ZNC@rGO (Fig. 4i) are due to the reflections of α-Ni(OH)_2_ and the reflections of ZnO disappear owing to the embedded alpha nickel hydroxide structure which is consistent with the results of SEM and XRD analysis.

### 3.2. Electrochemical performance

**Fig. 5:**
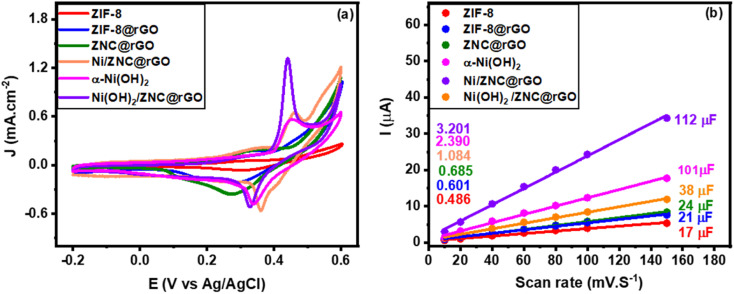
(a) CV curves recorded at 50 mV s^−1^ in 1 M KOH and (b) the corresponding linear fitting of the current (*i*) as a function of scan rates for the fabricated catalysts.

The electrochemical behavior of the fabricated catalysts was examined by the cyclic voltammetry (CV) in an alkaline solution (KOH) at a potential window ranging from −0.2 to 0.6 V with a scan rate of 50 mV s^−1^ (Fig. 5a). The figure shows that ZIF has very small redox peaks because of the low conductivity of MOFs.^36^ However, the ZIF-8@rGO current density increases compared to the pristine ZIF-8 owing to the ability of electron transfer, high conductivity, and large surface area of the rGO which enable the reactants to reach the ZnO species in the enclosed structure of ZIF leading to the oxidation of ethanol.^93^ Hence, the CV curves (Fig. 5a and S5a†) show a forward scan anodic peak at +0.32 V due to the reaction of Zn(ii) ions with OH^−^ to produce Zn(OH)_2_ followed by a small current peak (2) at +0.49 V owing to the oxidation of the Zn(OH)_2_ (Zn^2+^) to ZnOOH (Zn^3+^) according to eqn (5) and (6).^94^ Besides, a cathodic peak at +0.33 V is observed in the reverse scan due to the reduction of Zn^3+^ to Zn^2+^ (Fig. S5a†). Moreover, the CV curve of ZNC@rGO shows a higher current density with negative/positive shift of the anodic/cathodic peaks due to the conversion of the low conductive MOF to high conductive metal oxide-supported nitrogen-doped carbon matrix after the thermal treatment.

5 Zn + 2OH^−^ ↔ Zn(OH)_2_ + 2e^−^

6 Zn(OH)_2_ + OH^−^ ↔ ZnOOH + H_2_O + e^−^

The nickel-modified electrodes exhibit very high current density because of the synergistic effect of nickel or nickel hydroxide, ZnO, rGO, and nitrogen carbon matrix. It was observed that in the CV curve of Ni/ZNC@rGO (Fig. 5a and S5b†), the forward scan shows a small anodic peak current (1) at +0.32 V followed by a larger current peak (2) at +0.46 V owing to the oxidation of the Ni nanoparticles (Ni^0^) in β-Ni(OH)_2_ (Ni^2+^) and β-NiOOH (Ni^3+^), respectively according to eqn (7) and (8). Besides, a cathodic peak at +0.36 V is observed in the reverse scan due to the reduction of Ni^3+^ to Ni^2+^. Additionally, the electron donation to Ni(OH)_2_ from ZnO enhances the formation of primary NiOOH with high current density.

7 Ni + 2OH^−^ ↔ β-Ni(OH)_2_ + 2e^−^

8 β-Ni(OH)_2_ + OH^−^ ↔ β-NiOOH + H_2_O + e^−^

For pure α-Ni(OH)_2_, the CV curve shows a very prominent redox peak with an anodic peak at 0.38 V corresponding to the Ni^2+^ oxidation and another cathodic peak at 0.34 V related to the Ni^3+^ reduction according to eqn (9).^95^ The oxidation peak of α-Ni(OH)_2_ lies less positive than that for oxidation of β-Ni(OH)_2_ which formed from the oxidation of Ni nanoparticles. The observed differences in the CV of Ni/ZNC@rGO and Ni(OH)_2_ are due to the free ion/solvent transfer in the disordered α-Ni(OH)_2_/γ-NiOOH structures compared to the β-phase structures.^25^ The Ni(OH)_2_/ZNC@rGO catalyst exhibits the same CV curve as pure α-Ni(OH)_2_ with a remarkable increase in the current density due to the presence of high conductive rGO, the electron donation to α-Ni(OH)_2_ from ZnO, and the redox activity of the heteroatoms (N-atom).

9 α-Ni(OH)_2_ + OH^−^ ↔ NiOOH + H_2_O + e^−^

Moreover, the CV of the as-prepared catalysts was carried out at the non-faradaic region (0–0.1 V) at different scan rates ranging from 10 to 150 mV s^−1^ to determine the electrochemical surface area (ECSA). Fig. S6(a–f)† shows the CV curves of pure ZIF-8, ZIF-8@rGO, ZNC@rGO, Ni/ZNC@rGO, α-Ni(OH)_2_, and Ni(OH)_2_/ZNC@rGO, respectively, which exhibit an increase in the current of all materials at high scan rates. The double layer capacitance (*C*_dl_) can be estimated from the slope of the linear relationship of the current (at 0.10 V) and the applied scan rates (Fig. 5b). The obtained slopes of ZIF-8, ZIF-8@rGO, ZNC@rGO, Ni/ZNC@rGO, α-Ni(OH)_2_, and Ni(OH)_2_/ZNC@rGO precursors are found to be 17, 21, 24, 38, 101, and 112 μF and from eqn (1), the ECSA is found to be 0.48, 0.60, 0.69, 1.09, 2.39, and 3.20 cm^2^, respectively. From the obtained data, the Ni(OH)_2_/ZNC@rGO catalyst has the largest ECSA value relative to the other catalysts, and thus the highest catalytic activity which agrees with the results of CV.

### 3.3. Electrochemical oxidation of ethanol

The catalytic activity of the ZIF-8, ZIF-8@rGO, ZNC@rGO, Ni/ZNC@rGO, α-Ni(OH)_2_, and Ni(OH)_2_/ZNC@rGO electrocatalysts towards EOR was conducted by CV measurements in 1 M ethanol + 1 M KOH. Compared to the CV curves of these catalysts in the absence of ethanol, Fig. S7(a–f)† shows some changes in the CV in the presence of ethanol. For Ni(OH)_2_/ZNC@rGO catalyst (Fig. S7f†), the catalytic activity toward EOR is confirmed by the increase of the current density of the peak at 0.45 V related to the NiOOH formation, as well as the peak at 0.50 V in the forward scan which is attributed to the ethanol oxidation. In contrast, the current density of the cathodic peak (reverse scan) decreases in the presence of ethanol due to the decrease of NiOOH amount on the electrode surface which is consumed *via* the chemical reaction with ethanol oxidation (eqn (10)) instead of the electro-reduction reaction.^96^

10 NiOOH + EtOH ↔ Ni(OH)_2_ + products

**Fig. 6:**
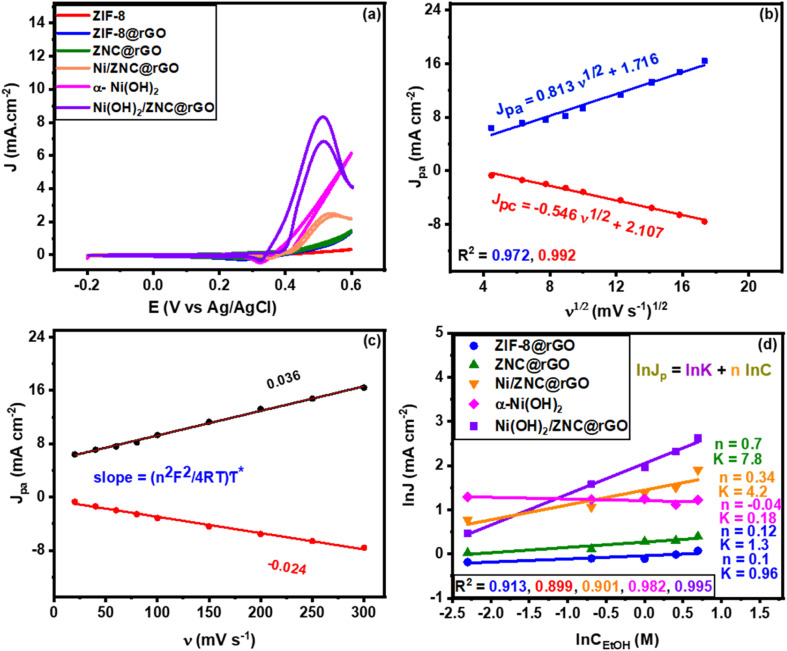
(a) CV curves of the modified electrodes in (1 M KOH + 1 M EtOH) at a scan rate of 50 mV s^−1^, (b) the linear relationship of anodic current (*I*_pa_) and cathodic current (*I*_pc_) densities *versus* the square root of scan rates, (c) the linear relationship of anodic current (*I*_pa_) and cathodic current (*I*_pc_) densities *versus* the scan rates for Ni(OH)_2_/ZNC@rGO, and (d) plots of ln *J*_p_*vs.* ln *C*_ethanol_ at constant potential obtained from Fig. S12.†

Fig. 6a and S8† show that the bare ZIF-8 cannot oxidize the ethanol, while it is oxidized well by the modified electrodes reaching the high activity by Ni(OH)_2_/ZNC@rGO with a negatively shifted anodic peak at 0.5 V with a current density of 8.3 mA cm^−2^ (31.9 A g^−1^) and an onset potential of 0.34 V. This may be due to the synergistic effect of the high diffusion of the electrons through the rGO sheets and the various oxidation states of ZnO and Ni(OH)_2_.^96^ Additionally, the structural defects resulting from the nitrogen doping increase the strong electron transfer among Ni(OH)_2_ and ZnO nanoparticles. Also, it is noticeable that the anodic oxidation peak of ethanol shifts toward more negative potentials compared to the previous literature as displayed in Table S1†, indicating that nickel hydroxide is necessary for electrocatalytic ethanol oxidation. Similarly, the pure α-Ni(OH)_2_ shows high activity for ethanol oxidation at an onset potential of 0.38 V with a current density of 6.2 mA cm^−2^. However, the onset potential of α-Ni(OH)_2_ is more positive than Ni(OH)_2_/ZNC@rGO composite indicating the role of the ZNC@rGO support on the development of the catalytic activity. Compared to the Ni(OH)_2_-based catalysts, the Ni/ZNC@rGO catalyst exhibits a more positive anodic peak at 0.55 V with an onset potential of 0.42 V and a low current density of 2.07 mA cm^−2^. The low activity of Ni/ZNC@rGO catalyst could be due to the oxidation of Ni nanoparticles to the more ordered and less active β-phase structure of Ni(OH)_2_/NiOOH couples.

Besides, the as-prepared catalysts were studied for EOR at different scan rates in an alkaline solution in the presence of 1 M EtOH. As shown in Fig. S9,† at high values of scan rate, the current densities of ethanol oxidation peak (forward scan) and the NiOOH reduction (reverse scan) increase for all catalysts as there was not enough time for the EtOH and NiOOH reaction and thus the redox reaction of Ni(OH)_2_/NiOOH is the major. Meanwhile, the linear relationship of the square root of the scan rate and the peak current density from ZIF-8@rGO, ZNC@rGO, Ni/ZNC@rGO, α-Ni(OH)_2_, and Ni(OH)_2_/ZNC@rGO are displayed in Fig. S10(a–d)† and 6b, respectively. The figures show a linear proportion indicating that the EOR on these modified electrodes is controlled by the diffusion process.^96^ Using the Randles–Sevcik equation (eqn (2)), the diffusion coefficients (*D*) were calculated for each sample and listed as follows; 0.25 × 10^−9^, 0.44 × 10^−9^, 1.02 × 10^−9^, 0.95 × 10^−9^, and 1.83 × 10^−7^ for ZIF-8@rGO, ZNC@rGO, Ni/ZNC@rGO, α-Ni(OH)_2_, and Ni(OH)_2_/ZNC@rGO, respectively. It reveals that compared with all prepared catalysts, the charge-transport rate within the interface between the electrolyte and the Ni(OH)_2_/ZNC@rGO electrode is the highest.

The influence of the modified electrode was also studied through the measurements of the surface coverage (τ*) by the redox species from the CV measurements at various scan rates. From the plots of the anodic and cathodic current densities *versus* the different scan rates for ZIF-8@rGO (Fig. S11a†), ZNC@rGO (Fig. S11b†), Ni/ZNC@rGO (Fig. S11c†), α-Ni(OH)_2_ (Fig. S11d†), and Ni(OH)_2_/ZNC@rGO (Fig. 6c), it is observed that all materials show a linear relationship. From this linear relationship, the τ* values were calculated using eqn (3) which are 0.11 × 10^−9^, 0.22 × 10^−9^, 12.7 × 10^−9^, 19.2 × 10^−9^, and 38.3 × 10^−9^ mol cm^−2^ for ZIF-8@rGO, ZNC@rGO, Ni/ZNC@rGO, α-Ni(OH)_2_, and Ni(OH)_2_/ZNC@rGO, respectively. It is observed that the thickness of Ni^2+^/Ni^3+^ species on the Ni(OH)_2_/ZNC@rGO surface is three times more than those of Ni/ZNC@rGO due to the fast oxidation of α-Ni(OH)_2_ compared to the most crystalline phase β-Ni(OH)_2_. Those results confirm that α-Ni(OH)_2_ played an important role in the EOR improvement. Moreover, the catalytic activity of EOR can be well-expressed using the turnover frequency (TOF) which is defined as the number of converted reactant molecules into product molecules in unit time per active site. The TOF can be calculated as reported in the literature using the following equation:^97,98^11
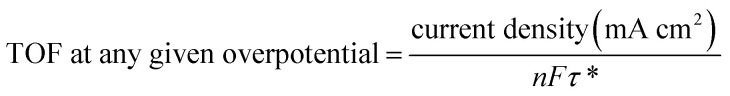
where *n* is the number of electrons of the complete oxidation of ethanol (12 e), *F* is the Faraday constant, and τ* is the calculated surface coverage. The TOFs are found to be 0.161, 0.163, 0.165, 0.168, and 0.189 s^−1^ for ZIF-8@rG, ZNC@rGO, Ni/ZNC@rGO, α-Ni(OH)_2_, and Ni(OH)_2_/ZNC@rGO, respectively, confirming the high activity of Ni(OH)_2_/ZNC@rGO composite for the oxidation of ethanol.

Since the catalytic oxidation of ethanol is affected by the concentration of ethanol, the EOR was performed with different ethanol concentrations. Fig. S12(a–e)† displays the typical CVs of ZIF-8@rGO, ZNC@rGO, Ni/ZNC@rGO, α-Ni(OH)_2_, and Ni(OH)_2_/ZNC@rGO, respectively, at a scan rate of 50 mV s^−1^ in different concentrations of EtOH (0.1–2 M). The figure displays an increase in the anodic current density with high ethanol concentrations due to the ethoxy adsorption on the surface of the catalyst. Meanwhile, the plot of ln current *vs.* ln ethanol concentration at a specific potential (Fig. 6d) is used to estimate the reaction orders as follows:^76^

12 ln *I*_p_ = ln *k* + *n* ln *C*_EtOH_

where *k* and *n* are the reactions constant and order concerning ethanol, respectively. The overall reaction orders are found to be 0.1, 0.12, 0.34, 0.04, and 0.7 with rate constants of 0.96, 1.3, 4.2, 0.18, and 7.8 s^−1^ for ZIF-8@rGO, ZNC@rGO, Ni/ZNC@rGO, α-Ni(OH)_2_, and Ni(OH)_2_/ZNC@rGO, respectively, which agree with other studies.^9^

Additionally, the catalytic oxidation of ethanol is affected by the type and concentration of the electrolyte. Thus, the CV technique of the as-prepared Ni(OH)_2_/ZNC@rGO was tested in alkaline (KOH, 1 M) and acidic (H_2_SO_4_, 1 M) electrolytes in the absence of ethanol (Fig. S13a†) and in the presence of 1 M ethanol (Fig. S13b†). The figures show that the catalytic activity of ethanol is highly effective in alkaline electrolytes (*E*_onset_ = 0.34 V) than the acidic electrolytes (*E*_onset_ = 0.53 V). Owing to the oxophilic nature of the catalysts, the alkaline medium facilitates the oxidation of the Ni(OH)_2_ to NiOOH active species which enhances the ethanol oxidation performance. Moreover, the effect of the concentration of the KOH electrolyte at the different electrodes was investigated in the presence of 1 M ethanol at a scan rate of 50 mV s^−1^. Fig. S14(a–e)† shows the CV of the different electrodes in a 1.0 M ethanol solution containing various concentrations of KOH solutions (0.1–1.0 M). It can be observed that the peak current density for all catalysts increases with the concentration of KOH indicating that the kinetics of the EOR are improved by the greater availability of OH^−^ ions in the solution. Meanwhile, the plot of ln current *vs.* ln KOH concentration at a specific potential (Fig. S14f†) is used to estimate the reaction orders using eqn (12). The overall reaction orders concerning electrolytes are found to be 0.85, 1.15, 0.37, 0.34, and 1.4 with rate constants of 0.35, 0.71, 0.1, 0.47, and 0.74 s^−1^ for ZIF-8@rGO, ZNC@rGO, Ni/ZNC@rGO, α-Ni(OH)_2_, and Ni(OH)_2_/ZNC@rGO, respectively.

### 3.4. Kinetic investigation of EOR

**Fig. 7:**
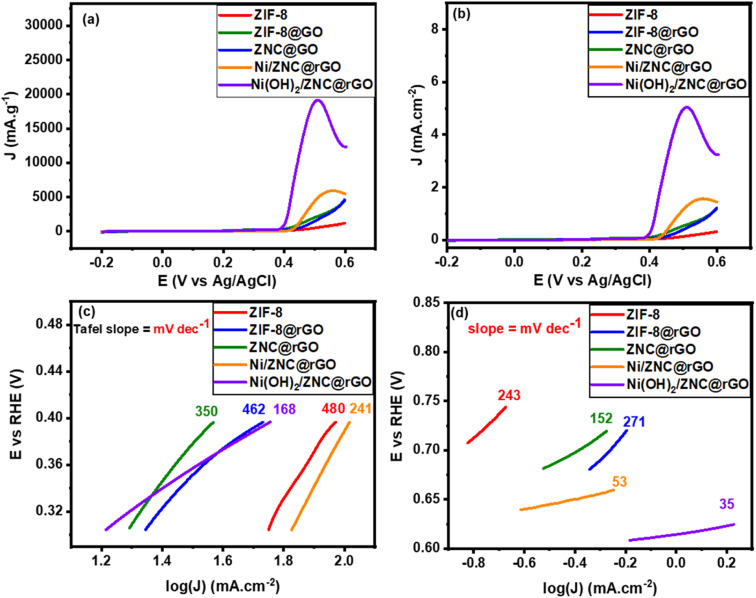
LSV curves of EOR at a low scan rate of 10 mV s^−1^ in 1 M KOH + 1 M ethanol solution, (a) mass activity and (b) specific activity and Tafel plots of the EOR at a potential range (c) below and (d) above the onset potential.

Additionally, the LSV technique was performed at a scan rate of 10 mV s^−1^ for the as-fabricated catalysts in N_2_-saturated KOH (1 M) and EtOH (1 M) solution. In the potential region below 0.35 V *vs.* Ag/AgCl, the mass activity (Fig. 7a) and specific activity (Fig. 7b) show a slow increase in the current density of all electrocatalysts with the increase of potential. While, at the potential region above 0.35 V, the current increases sharply at high potential reaching the highest value using the Ni(OH)_2_/ZNC@rGO catalyst. The Tafel plot is a useful technique for studying the kinetics of reactions and mechanisms of modified electrodes. The Tafel slopes were derived from the linear region of the potential *versus* log *J* plot as follows;^9,99^

13 *η* = *a* + *b* log *i*

where *η* (V), *a*, *b*, and *i* (mA cm^−2^) are the overpotential, the Tafel slope, the Tafel intercept, and the current density, respectively.

The first slope values of all catalysts were calculated from the linear region of the LSV below the *E*_onset_ of EOR and found to be 480, 462, 350, 241, and 168 mV dec^−1^ for ZIF-8, ZIF-8@rGO, ZNC@rGO, Ni/ZNC@rGO, and Ni(OH)_2_/ZNC@rGO, respectively (Fig. 6c). Based on the lowest Tafel slope of Ni(OH)_2_/ZNC@rGO, the charge transfer kinetics on this catalyst are faster than on the other ethanol oxidation catalysts. Furthermore, the second Tafel slopes were determined from the potential plot of the linear region above the *E*_onset_ of ethanol oxidation and are 243, 271, 152, 53, and 35 mV dec^−1^, respectively (Fig. 6d). The lower values of the second Tafel slopes compared to the first slopes values show that the ethanol oxidation mechanism has changed.

Based on the Tafel slopes, constructive information on the mechanism of EOR can be investigated using the potentiodynamic pseudo steady state polarization using the following equations:^9^14
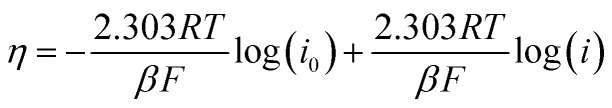
where *β*, *i*_o_, *R*, *F*, and *T* are the charge transfer coefficient, the exchange current density, the ideal gas constant, the Faraday constant, and the absolute temperature in K, respectively.

Meanwhile, the extrapolating of the Tafel line to the overpotential of zero can be used to calculate the exchange current densities (*i*_0_). The kinetic parameters, equilibrium charge transfer coefficients (*β*), and exchange current density (*i*_0_), are represented in Table 1. A higher value of *i*_0_ in the electrochemical reactions indicates a faster reaction of the modified electrode, which is the key to effective electrocatalysts and thus good ethanol oxidation performance. The highest (*i*_0_) value is achieved on Ni(OH)_2_/ZNC@rGO electrode (20.9 mA cm^−2^) relative to that of ZIF-8 (3.75 mA cm^−2^), ZIF-8@rGO (9.28 mA cm^−2^), ZNC@rGO (6.39 mA cm^−2^), and Ni/ZNC@rGO (18.62 mA cm^−2^). Also, the anodic transfer coefficient has a value ranging from 0 to 1, which is an important measure in the Tafel equation as it signifies the activation barrier for electrooxidation reactions. The high value of the anodic transfer coefficient for Ni(OH)_2_/ZNC@rGO electrode indicates that the electrode is a sufficient electrocatalyst to enhance the EOR and has greater kinetics than others.

**Table 1:** Comparison list of electrocatalytic parameters of the fabricated catalysts for the EOR

Sample	ECSA (cm^2^)	*C*_dl_ (μF)	τ* (mol cm^−2^)	Tafel slope (mV dec^−1^)	*i*_0_ (mA cm^−2^)	*β*	TOF (s^−1^)
ZIF-8	0.48	17	—	271	3.75	0.22	—
ZIF-8@rGO	0.60	21	0.11 × 10^−9^	243	4.28	0.25	0.161
ZNC@rGO	0.69	24	0.22 × 10^−9^	152	6.39	0.39	0.163
Ni/ZNC@rGO	1.09	38	12.7 × 10^−9^	53	18.62	1.18	0.165
α-Ni(OH)_2_	2.39	101	19.2 × 10^−9^	—	—	—	0.168
Ni(OH)_2_/ZNC@rGO	3.20	112	38.3 × 10^−9^	35	20.91	1.52	0.189

### 3.5. EIS measurements

**Fig. 8:**
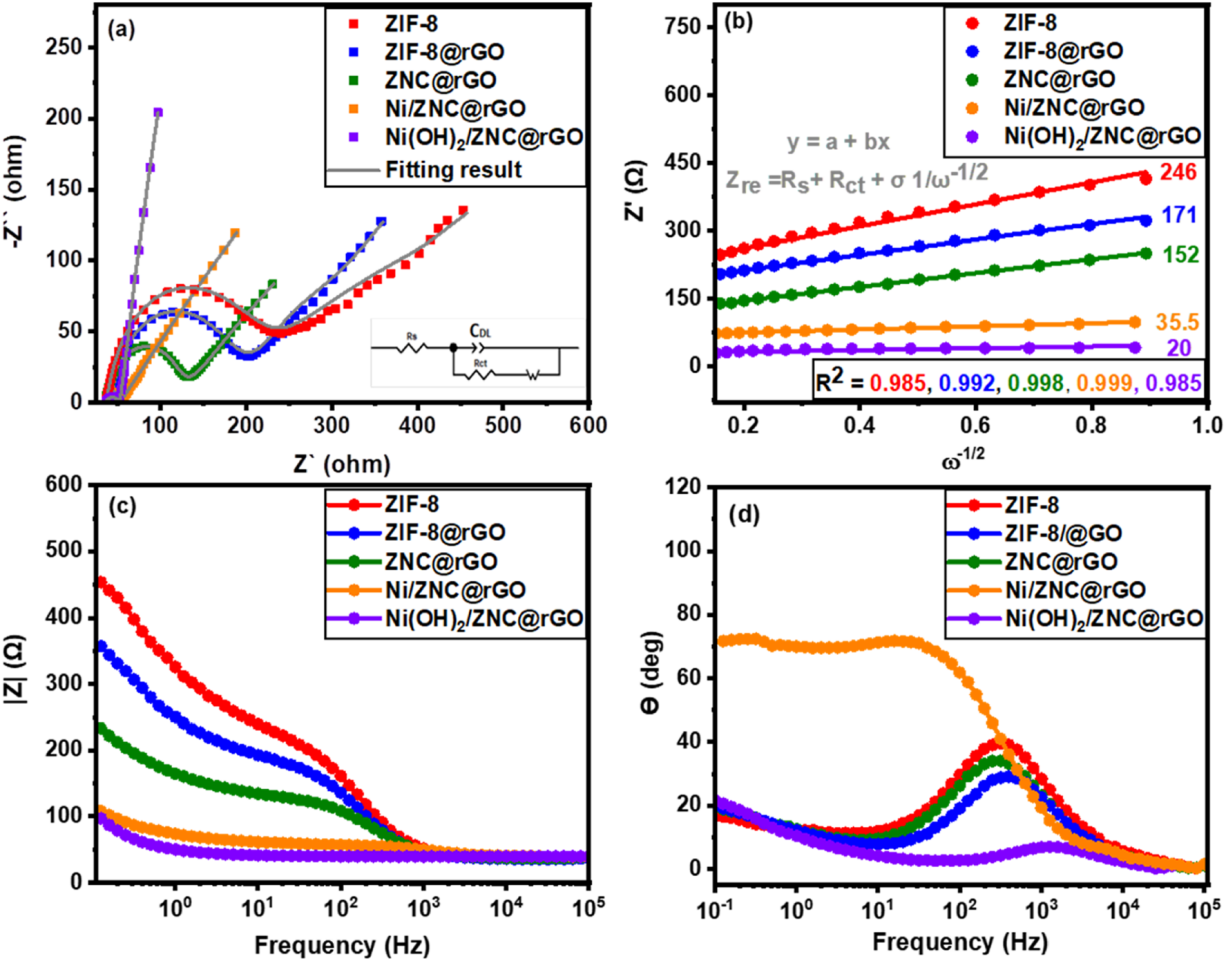
(a) The EIS plots of the as-prepared catalysts at 0.6 V *vs.* Ag/AgCl and an amplitude of 10 mV in 1 M KOH + 1 M EtOH solution, (b) the relationship of *Z*_re_*vs. ω*^−1/2^, (c) the frequency-dependent real part of the impedance, and (d) Bode plots for all catalysts.

The EIS approach is used to assess the validity of the as-fabricated catalysts' resistance and ion transport/diffusion capability at the electrolyte interface with the modified electrode surface.^100^ The Ni(OH)_2_/ZNC@rGO electrode has a smaller semicircle diameter than the other electrocatalysts, as shown in Fig. 8a and Table 2, demonstrating the reduced resistance of the charge transfer for EOR and higher kinetics on the surface. Moreover, the catalyst exhibits faster electron transfer during the EOR which is confirmed by the smaller value of the charge transfer resistance (*R*_ct_) achieving the best electron conductivity and ions transport compared to the other prepared electrocatalysts. These results are related to the high surface area of the rGO and the derived carbon. Besides, the rGO and N-doped carbon exhibit high conductivity which promotes the electron transfer process.^100^ The experimental data of all as-prepared catalysts are fitted to standard Randles equivalent circuits for ethanol electrooxidation (inset of Fig. 8a), which comprises the resistance of the charge transfer resistance (*R*_ct_), the resistance of the solution (*R*_s_), and the *C*_dl_ for the electrodes. Moreover, Fig. 8b displays the linear relationship of the impedance real part (*Z*_re_) *versus* the reciprocal of the square root of the angular frequency (*ω*^−1/2^) recorded at the region of low-frequency which is used to confirm the conductivity of electrodes. Also, the diffusion coefficient (*D*) of the OH^−^ was calculated using the following equations:^101^

15 *Z*_re_ = *R*_ct_ + *R*_s_ + *σω*^−1/2^

16 *D* = *R*^2^*T*^2^/(2*A*^2^*F*^4^*C*^2^*n*^4^*σ*^2^)

where *R*_ct_, *σ*, and *R*_s_ are the charge transfer resistance, Warburg factor (the slope of the linear relationship in Fig. 8b), and inner resistance, respectively. Also, *R*, *F*, *n*, *T*, *A*, and *C* are the ideal gas constant (8.314 J mol^−1^ K^−1^), the Faraday constant (96 486 C mol^−1^), the number of electrons per molecule, the absolute temperature (K), the surface area of the GC electrode (7.065 cm^2^), and the concentration of the ions (1 × 10^−3^ mol cm^−3^), respectively. Resultantly, the *D* value is found to be about 1.68 × 10^−7^ cm^2^ s^−1^ for the Ni(OH)_2_/ZNC@rGO electrode which is higher than the values of ZIF-8 (0.03 × 10^−9^ cm^2^ s^−1^), ZIF-8@rGO (0.07 × 10^−9^ cm^2^ s^−1^), ZNC@rGO (0.09 × 10^−9^ cm^2^ s^−1^), and Ni(OH)_2_/ZNC@rGO (5.23 × 10^−7^ cm^2^ s^−1^).

**Table 2:** Comparison list of EIS parameters of the fabricated catalysts for the EOR

Sample	*R*_s_ (Ω)	*R*_ct_ (Ω)	*θ* (°)
ZIF-8	38	209.1	8
ZIF-8@rGO	41	159.2	29
ZNC@rGO	38	95.4	34
Ni/ZNC@rGO	42	45.1	71
Ni(OH)_2_/ZNC@rGO	39	20.8	40

Additionally, Fig. 8c represents a relation of the real part of impedance (*Z*′) *versus* the frequency for the as-fabricated electrodes. The figure shows that *Z*′ decreases with the increase in frequency for all the samples. However, the magnitude value of *Z*′ for Ni(OH)_2_/ZNC@rGO is the smallest indicating the low electrode interface resistances. Finally, the kinetics of electron diffusion can be investigated from the Bode plots at a low frequency of 10^5^ Hz (Fig. 8d) which display the phase angle values of 40°, 34°, 29°, 71°, and 8° for ZIF-8, ZIF-8@rGO, ZNC@rGO, Ni/ZNC@rGO, and Ni(OH)_2_/ZNC@rGO electrodes, respectively. The low value of the phase angle indicates the high diffusion of the electrons during the EOR.^102^ The obtained results indicate that α-Ni(OH)_2_ exhibits faster kinetics compared to β-Ni(OH)_2_ produced by the oxidation of Ni nanoparticles in the alkaline medium.

### 3.6. Effect of temperature on the EIS measurements

**Fig. 9:**
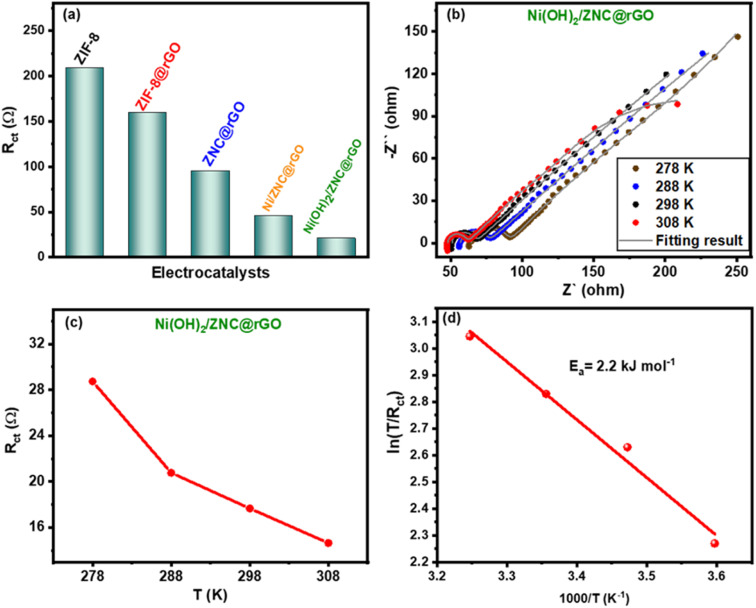
Comparison of charge transfer resistance (*R*_ct_) for all catalysts (a), EIS plots of the Ni(OH)_2_/ZNC@rGO electrode at various temperatures (5, 15, 25, and 35 °C) (b), comparative charge transfer resistance for Ni(OH)_2_/ZNC@rGO at various temperatures (c), and plot of ln(*T*/*R*_ct_) and 1000/*T* for Ni(OH)_2_/ZNC@rGO estimating the activation energy (*E*_a_) (d).

As mentioned above, the EIS measurements were performed for all catalysts to determine the resistance values of the charge transfer (*R*_ct_). From Fig. 9a, the Ni(OH)_2_/ZNC@rGO electrode possesses the lowest value of *R*_ct_ (20.8 Ω) compared to ZIF-8 (209.1 Ω), ZIF-8@rGO (159.2 Ω), ZNC@rGO (95.4 Ω), and Ni/ZNC@rGO (45.1 Ω). Additionally, the EIS data were recorded for the Ni(OH)_2_/ZNC@rGO electrode at various temperatures (5, 15, 25, and 35 °C) to estimate the activation energy (*E*_a_) (Fig. 9b). The figure displays that the semicircle at high frequencies and thus the value of *R*_ct_ decreases at high temperatures due to the ease of the electrochemical kinetic reactions. The corresponding *R*_ct_ values were calculated and displayed in Fig. 9c which are found to be 28.7, 20.8, 17.7, and 14.6 Ω at 5, 15, 25, and 35 °C, respectively. Based on these data, the activation energy (*E*_a_) was estimated as follows;^103^

17 *i*_0_ = *RT*/*nFR*_ct_

18 *i*_0_ = *A* exp(−*E*_a_/*RT*)

where *A* is the temperature-independent coefficient. According to the Arrhenius equation, the linear relation of ln(*T*/*R*_ct_) and 1000/*T* gives a slope equal to the activation energy (Fig. 9d) which is found to be very low (2.2 kJ mol^−1^) confirming an easier diffusion process for EOR.

### 3.7. Long-term stability performances

**Fig. 10:**
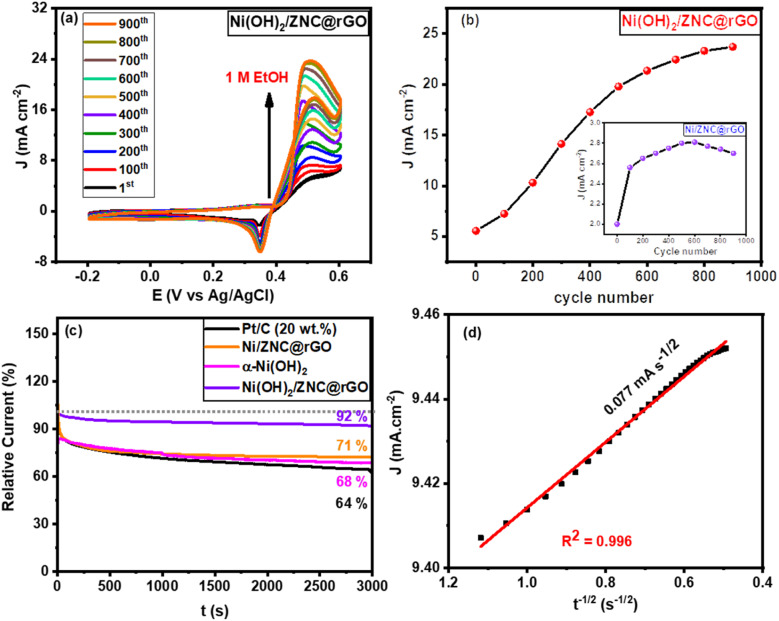
Cycling stability tests of the prepared catalysts (a) CVs curves for 900 cycles from −0.2–0.6 V at a scan rate of 10 mV s^−1^ in 1 M KOH + 1 M EtOH using Ni(OH)_2_/ZNC@rGO, (b) the relation of the EOR current density *versus* the cycle number for Ni(OH)_2_/ZNC@rGO (the inset is for Ni/ZNC@rGO), (c) *I*–*t* curve of the prepared catalysts for 1 M ethanol electrooxidation, and (d) the plot of the current density and the square root of the electrooxidation time.

To further confirm the high activity of the Ni/ZNC@rGO and Ni(OH)_2_/ZNC@rGO catalysts over an extended period, the CV technique was performed for consecutive hour-long periods over the potential window. As shown in Fig. 10a and S15,† the CV technique was tested for 900 cycles at a potential of −0.2–0.6 V in 1 M KOH containing 1 M EtOH using Ni/ZNC@rGO and Ni(OH)_2_/ZNC@rGO, respectively. It is observed that the Ni(OH)_2_/ZNC@rGO catalyst can maintain a stable potential with a continuous increase in the current density of the EOR (Fig. 10a) highlighting the remarkable activity and catalytic stability due to the increase of the redox species Ni^2+/3+^ on the surface. While Ni/ZNC@rGO catalyst exhibits stable current density till the 600th cycle followed by a decrease in the current density with further consecutive periods (Fig. S15†). From Fig. 10b, the current density of the 900th cycle (23.7 mA cm^−2^) using Ni(OH)_2_/ZNC@rGO increases by 4.3 times of the 1st cycle (5.6 mA cm^−2^). In contrast, the current density of Ni/ZNC@rGO catalyst after 600th is 1.4 times that of the 1st cycle and then decreases by further consecutive stability cycles (inset Fig. 10b).

Besides, the chronoamperometric responses of the Ni(OH)_2_/ZNC@rGO, Ni/ZNC@rGO, α-Ni(OH)_2_, and commercial Pt/C (20 wt%) electrodes were recorded for 3000 s at a constant potential (0.6 V) in an alkaline solution containing ethanol. Fig. 10c shows a rapid decrease in the current density of ethanol oxidation at the initial step owing to the rapid ethanol consumption at the catalyst and solution interface and the double-layer charging process. Afterwards, the current density becomes relatively stable with time when the ethanol molecules reach the catalyst surface with a constant value of the current density for the EOR.^100^ The current density of EOR at the Ni(OH)_2_/ZNC@rGO remained at 92% after 3000 s compared with the original value which is higher than that of the commercial 20 wt% Pt/C (64%) indicating the high stability of the prepared electrode (Fig. 10c). This might be owing to the high conductivity, and huge surface area of the ESI† (rGO and N-doped carbon) as well as the high activity of α-Ni(OH)_2_ and ZnO nanoparticles.^100^ On the other hand, the current density of the Ni/ZNC@rGO for EOR decreases to 71% after 3000 s compared with the original value. The low stability of the Ni/ZNC@rGO could be due to the slow reduction of the β-NiOOH which adsorbed on the catalyst surface and reduces the active sites. For α-Ni(OH)_2_, the current density decreased to 68% after 3000 s which is less than that of the ZNC@rGO-based catalyst indicating the role of the N-doped carbon and rGO in decreasing CO adsorption energy and thus enhancing the electrochemical stability. Finally, the diffusion coefficient was estimated from the slope of the linear relationship of the current density and the square root of the EOR time (Fig. 10d) which was found to be 4.01 × 10^−7^ cm^2^ s^−1^ using Cottrell law (eqn (4)).

## 4. Conclusion

In summary, the pyrolysis of MOFs to MOFs-derived N-doped carbon materials at high temperatures is a promising strategy for fabricating superactive electrocatalysts for EOR. The ZNC@rGO catalyst was fabricated through the thermal treatment of ZIF-8@rGO at 700 °C under an N_2_ atmosphere as a non-precious support for the Ni-based ctalysts. The support was loaded by the disorderd α-Ni(OH)_2_ nanoparticles *via* the facile refluxing method and Ni NPs *via* the hydrothermal method. To evaluate the activity of the electrocatalysts, CVs were applied from −0.2 to 0.6 V (*vs.* Ag/AgCl) at scan rates 50 mV s^−1^ in 1 M KOH and 1 M ethanol. The EOR onset potential of Ni(OH)_2_/ZNC@rGO electrode is strongly shifted to negative potential with the higher current value, compared to the other fabricated electrodes. The shift in the potential is due to the carbon-based material and ZnO nanoparticles which promote the OH^−^ adsorption at low potentials facilitating the active site formation (γ-NiOOH). Besides, the large interlayer spacing of α-Ni(OH)_2_ facilitates the ion-solvent intercalation. The Ni/ZNC@rGO electrocatalyst exhibited more positive onset potential due to the conversion of the Ni nanoparticles to more ordered β-phase Ni(OH)_2_ through potential cycling in alkaline media which slowly converted to the less active β-NiOOH species. Besides, the catalyst displays a high stability of 92% after 900 cycles relative to the Ni/ZNC@rGO and the commercial Pt/C catalysts. Finally, this work highlights the potential of fabricating highly efficient active and stable catalyst-derived MOFs for EOR applications using nonprecious-based materials.

## Conflicts of interest

The authors declare no competing financial interest.

## Supplementary Material

RA-014-D3RA08208C-s001
