# Supplementary material for: A comparative study of α-Ni(OH)2 and Ni nanoparticle supported ZIF-8@reduced graphene oxide-derived nitrogen doped carbon for electrocatalytic ethanol oxidation
Source: RSC Adv. 2024 Feb 13;14(8):5524–41. doi: 10.1039/d3ra08208c (PMC10863423; doi:10.1039/d3ra08208c)
Supplement: RA-014-D3RA08208C-s001 [file RA-014-D3RA08208C-s001.pdf]

Electronic Supplementary Material (ESI) for RSC Advances.
This journal is © The Royal Society of Chemistry 2024

# A comparative study of α-$Ni(OH)_2$ and Ni nanoparticles supported ZIF-8@reduced graphene oxide- derived nitrogen doped carbon for electrocatalytic ethanol oxidation

**Soliman Gamal, Doaa A Kospa, Amr Awad Ibrahim*, Awad I. Ahmed, A.M.A. Ouf**

*Department of Chemistry, Faculty of Science, Mansoura University, Al-Mansoura 35516, Egypt.*

## Supplementary data

**Fig. S1:** (a) $N_2$ adsorption-desorption isotherms and (b) the corresponding distributions of pore diameters of ZIF-8, ZIF@rGO, ZNC@rGO, and $Ni(OH)_2$/ZNC@rGO obtained from the desorption branch using the Barrett–Joyner–Halenda (BJH) method.

**Fig. S2: XPS of GO,** (a) Total survey and high-resolution spectra of (b) C1s and (c) O1s.

**Fig. S3:** (a) High magnification SEM image and (b) SEM of elemental mapping region of $Ni(OH)_2$/ZNC@rGO, and (c) EDX mapping of $Ni(OH)_2$/ZNC@rGO, (d) C, (e) O, (f) N, (g) Zn, and (h) Ni.

**Fig. S4:** EDX analysis of $Ni(OH)_2$/ZNC@rGO.

**Fig. S5:** CV curves of (a) ZIF-8, ZIF-8@rGO, and ZNC@rGO and (b) Ni/ZNC@rGO, α-$Ni(OH)_2$, and $Ni(OH)_2$/ZNC@rGO recorded at 50 mV $s^{-1}$ in 1M KOH.

**Fig. S6:** CV curves recorded in 1M KOH in the potential range of 0- 0.1 V at different scan rates for (a) ZIF-8, (b) ZIF-8@rGO, (c) ZNC@rGO, (d) Ni/ ZNC@rGO, (e) α$Ni(OH)_2$, and (f) $Ni(OH)_2$/ ZNC@rGO.

**Fig. S7:** CV curves of (a) ZIF-8, (b) ZIF-8@rGO, (c) ZNC@rGO, (d) Ni/ ZNC@rGO, (e) α-$Ni(OH)_2$ and (f) $Ni(OH)_2$/ ZNC@rGO in the absence and presence of 1M ethanol at a scan rate of 50 mV $s^{-1}$.

**Fig. S8:** CV curve of the as-prepared catalysts in 1M KOH + 1M Ethanol at a scan rate of 50 mV $s^{-1}$.

**Fig. S9:** CV curves recorded in 1M KOH in the potential range of -0.2- 0.6 V at different scan rates for (a) ZIF-8@rGO, (b) ZNC@rGO, (c) Ni/ ZNC@rGO, (d) α-$Ni(OH)_2$, and (e) $Ni(OH)_2$/ ZNC@rGO catalysts.

**Fig. S10:** Linear relationship of anodic ($I_{pa}$) and cathodic current ($I_{pc}$) densities of (a) ZIF-8@rGO, (b) ZNC@rGO, (c) Ni/ZNC@rGO, and (d) α-$Ni(OH)_2$ versus the square root of scan rates.

**Fig. S11:** Linear relationship of anodic ($I_{pa}$) and cathodic current ($I_{pc}$) densities of (a) ZIF-8@rGO, (b) ZNC@rGO, (c) Ni/ZNC@rGO, and (d) α-$Ni(OH)_2$ versus the scan rates.

**Fig. S12:** CV of (a) ZIF-8@rGO, (b) ZNC@rGO, Ni/ ZNC@rGO, (d) α-$Ni(OH)_2$ and (e) $Ni(OH)_2$/ ZNC@rGO catalysts in 1M KOH with different concentrations of ethanol (0.1- 2.0 M).

**Fig. S13**: CV of $Ni(OH)_2$/ ZNC@rGO in (a) 1M of alkaline and acidic electrolyte and (b) 1M of EtOH+ 1M of alkaline or acidic medium.

**Fig. S14:** CV of (a) ZIF-8@rGO, (b) ZNC@rGO, (c) Ni/ ZNC@rGO, (d) α-$Ni(OH)_2$ and (e) $Ni(OH)_2$/ ZNC@rGO catalysts in 1M EtOH with different concentrations of KOH electrolyte (0.1- 2.0 M) and (f) effect of concentration of the KOH electrolyte in electrocatalytic ethanol oxidation.

**Fig. S15:** CVs curves for 900 cycles from -0.2- 0.6 V at a scan rate of 10 mV $s^{-1}$ in 1M KOH+ 1M EtOH using Ni/ ZNC@rGO.

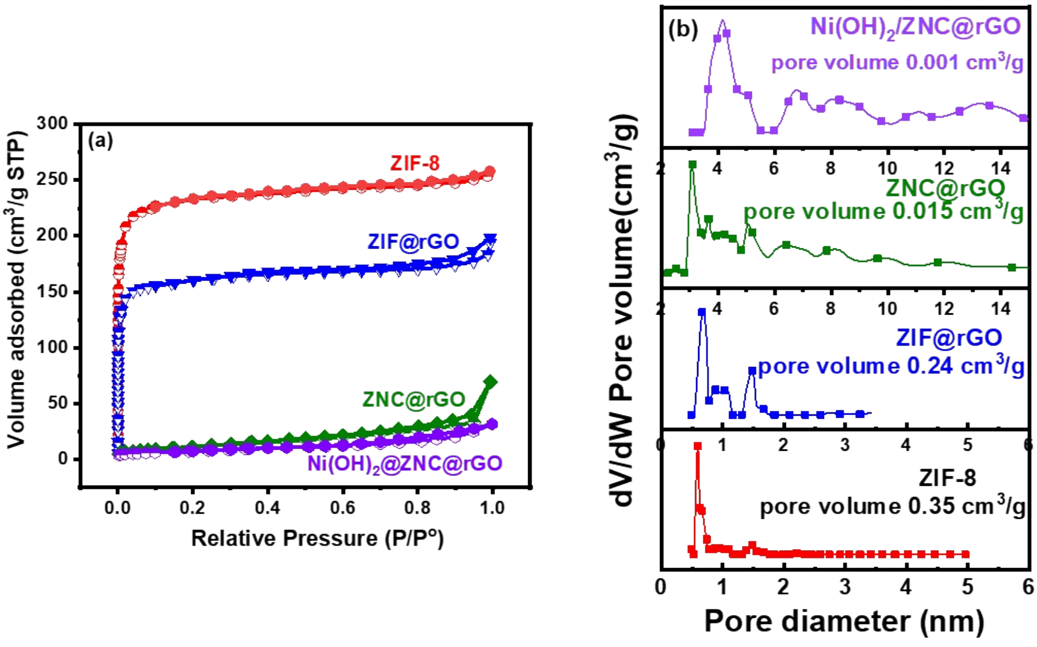


**Fig. S1:** (a) $N_2$ adsorption-desorption isotherms and (b) the corresponding distributions of pore diameters of ZIF-8, ZIF@rGO, ZNC@rGO, and $Ni(OH)_2$/ZNC@rGO obtained from the desorption branch using the Barrett–Joyner–Halenda (BJH) method.

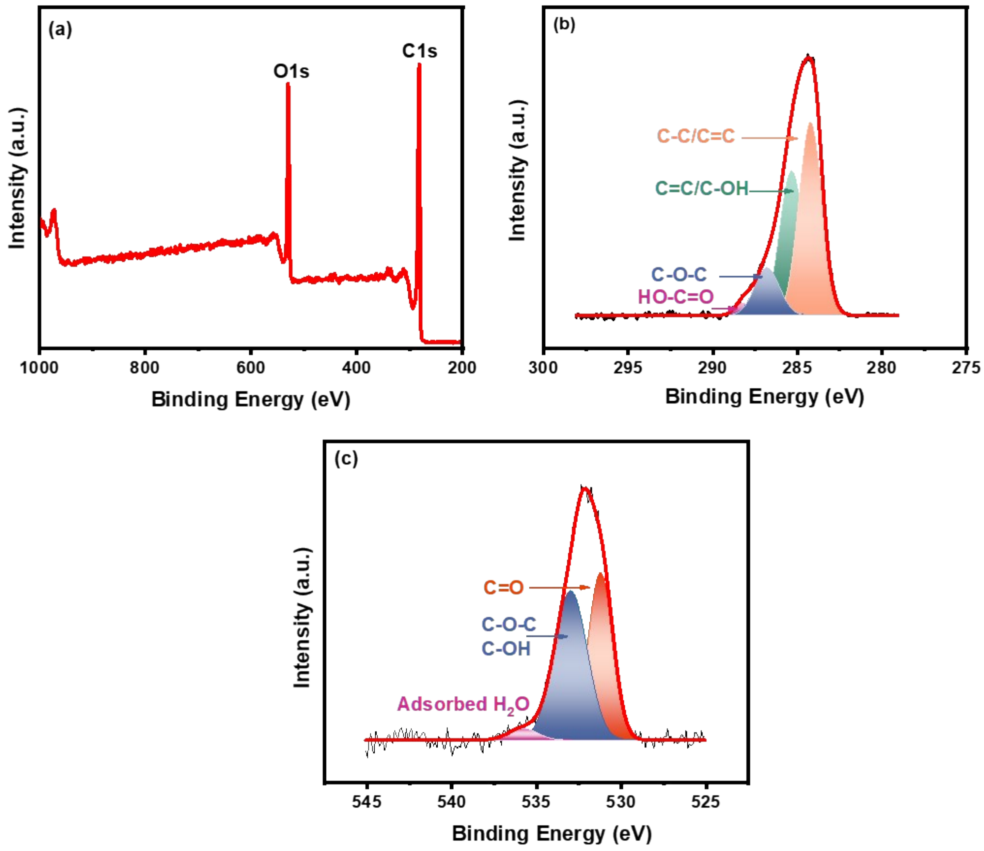


**Fig. S2: XPS of GO,** (a) Total survey and high-resolution spectra of (b) C1s and (c) O1s.

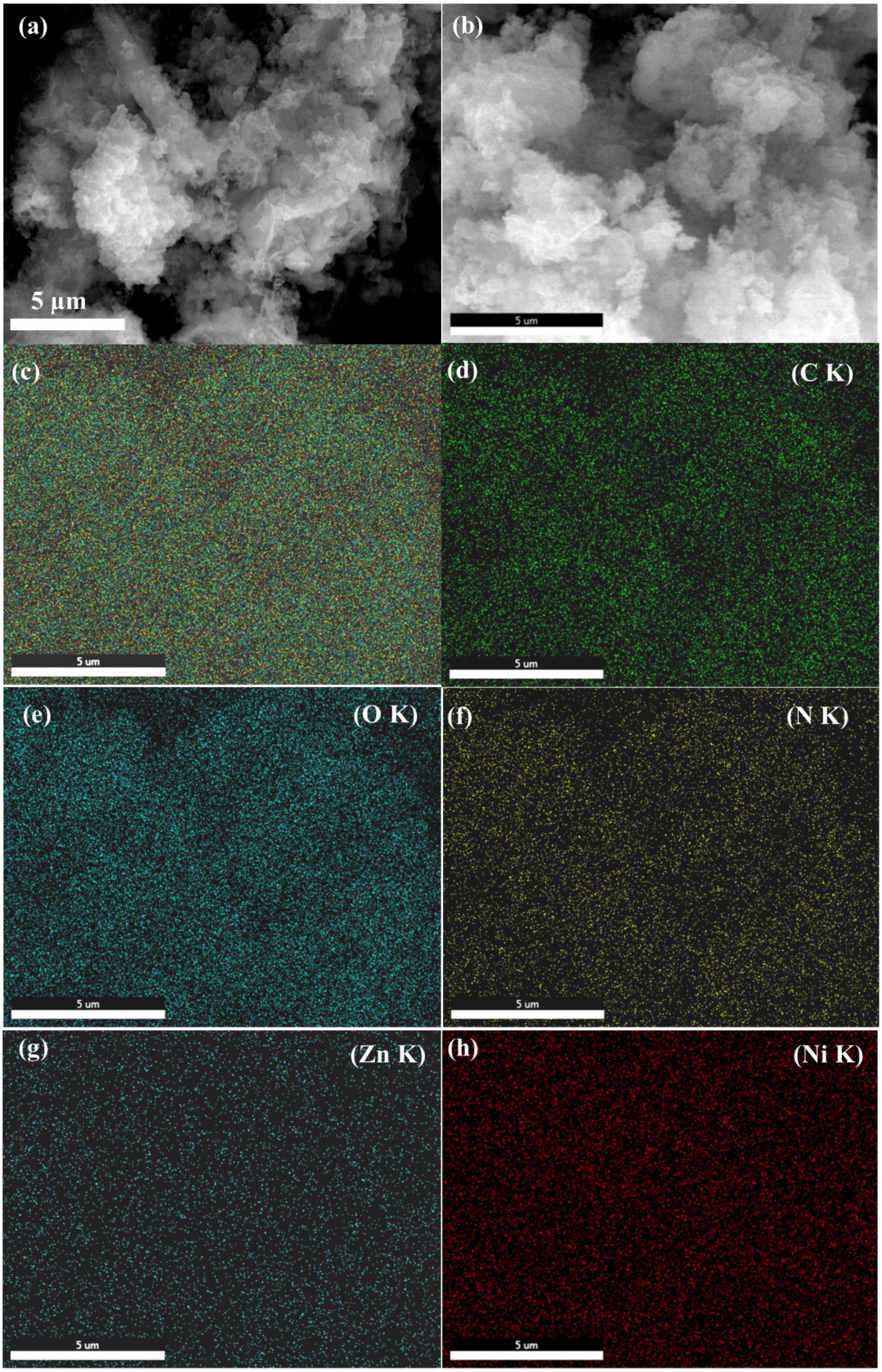


**Fig. S3:** (a) High magnification SEM image and (b) SEM of elemental mapping region of $Ni(OH)_2$/ZNC@rGO, and (c) EDX mapping of $Ni(OH)_2$/ZNC@rGO, (d) C, (e) O, (f) N, (g) Zn, and (h) Ni.

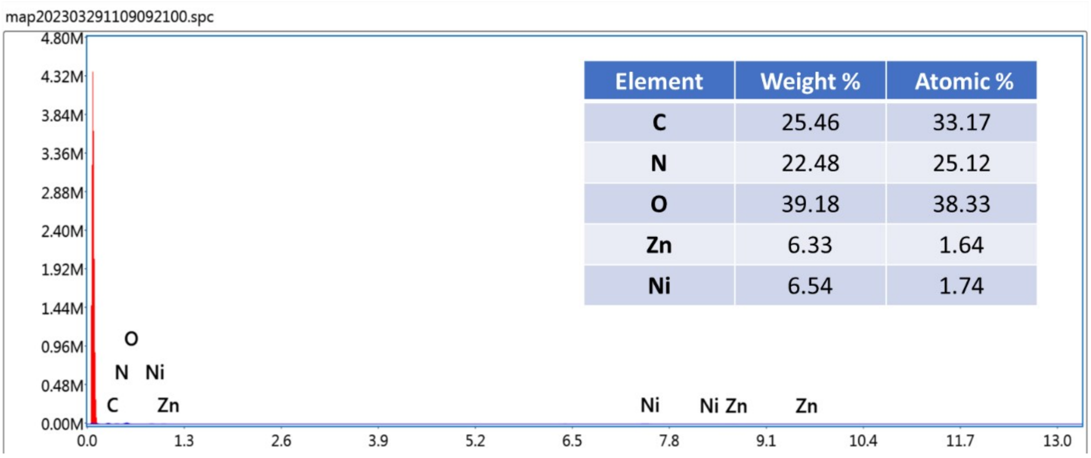


| Element | Weight % | Atomic % |
|---|---|---|
| C | 25.46 | 33.17 |
| N | 22.48 | 25.12 |
| O | 39.18 | 38.33 |
| Zn | 6.33 | 1.64 |
| Ni | 6.54 | 1.74 |

**Fig. S4:** EDX analysis of $Ni(OH)_2$/ZNC@rGO catalyst.

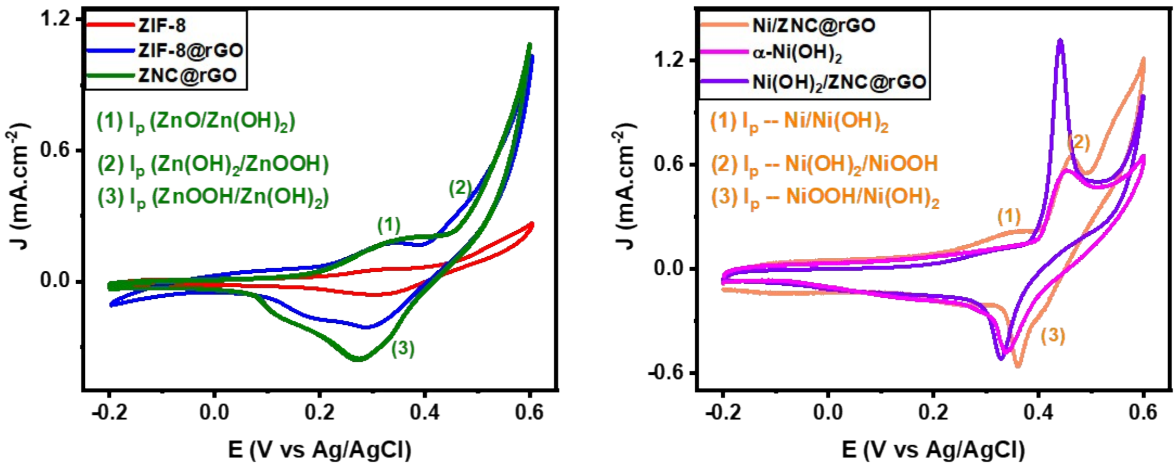


**Fig. S5:** CV curves of (a) ZIF-8, ZIF-8@rGO, and ZNC@rGO and (b) Ni/ZNC@rGO, α-$Ni(OH)_2$, and $Ni(OH)_2$/ZNC@rGO recorded at 50 mV $s^{-1}$ in 1M KOH.

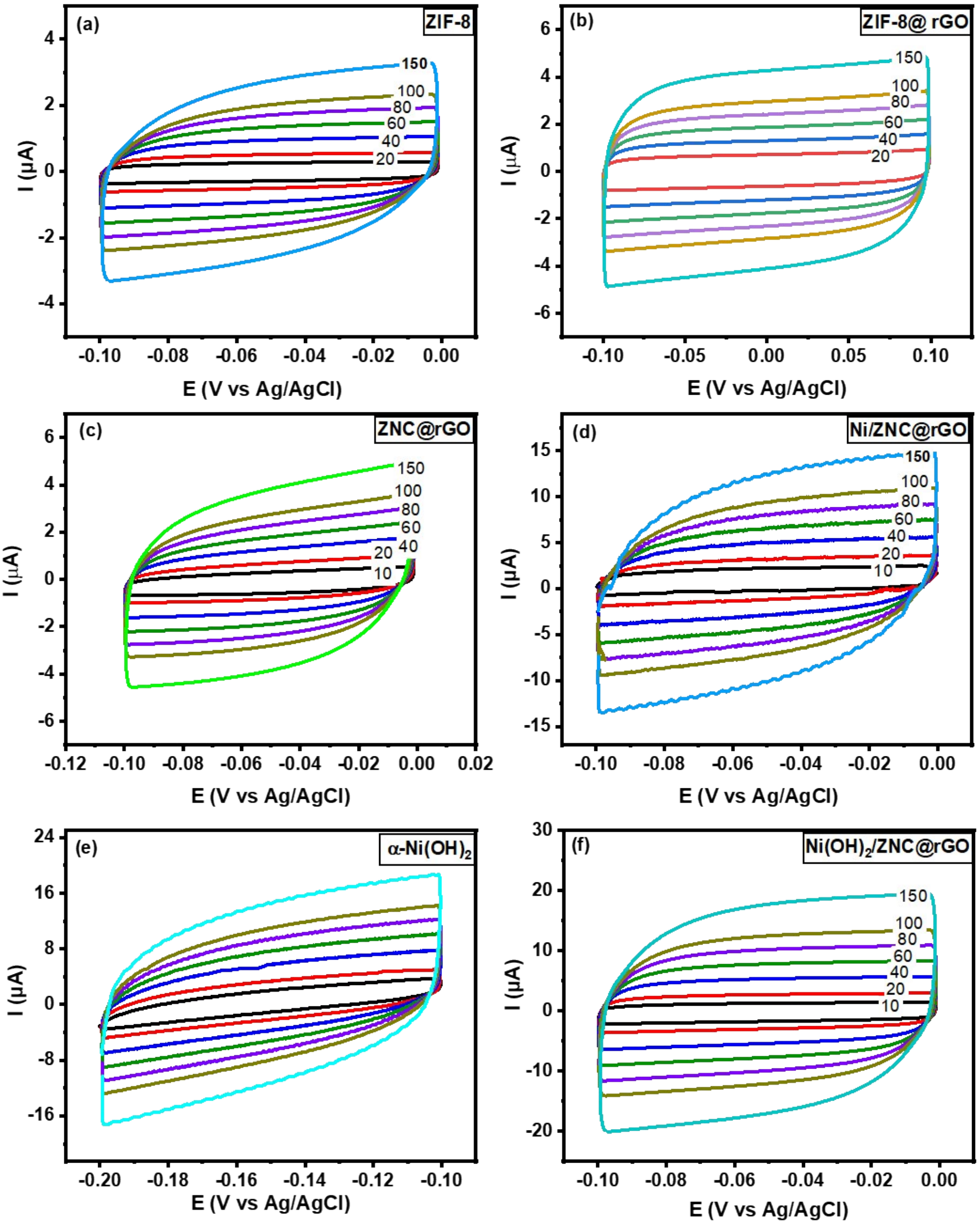


**Fig. S6:** CV curves recorded in 1M KOH in the potential range of 0- 0.1 V at different scan rates for (a) ZIF-8, (b) ZIF-8@rGO, (c) ZNC@rGO, (d) Ni/ ZNC@rGO, (e) αNi$(OH)_2$, and (f) Ni$(OH)_2$/ ZNC@rGO.

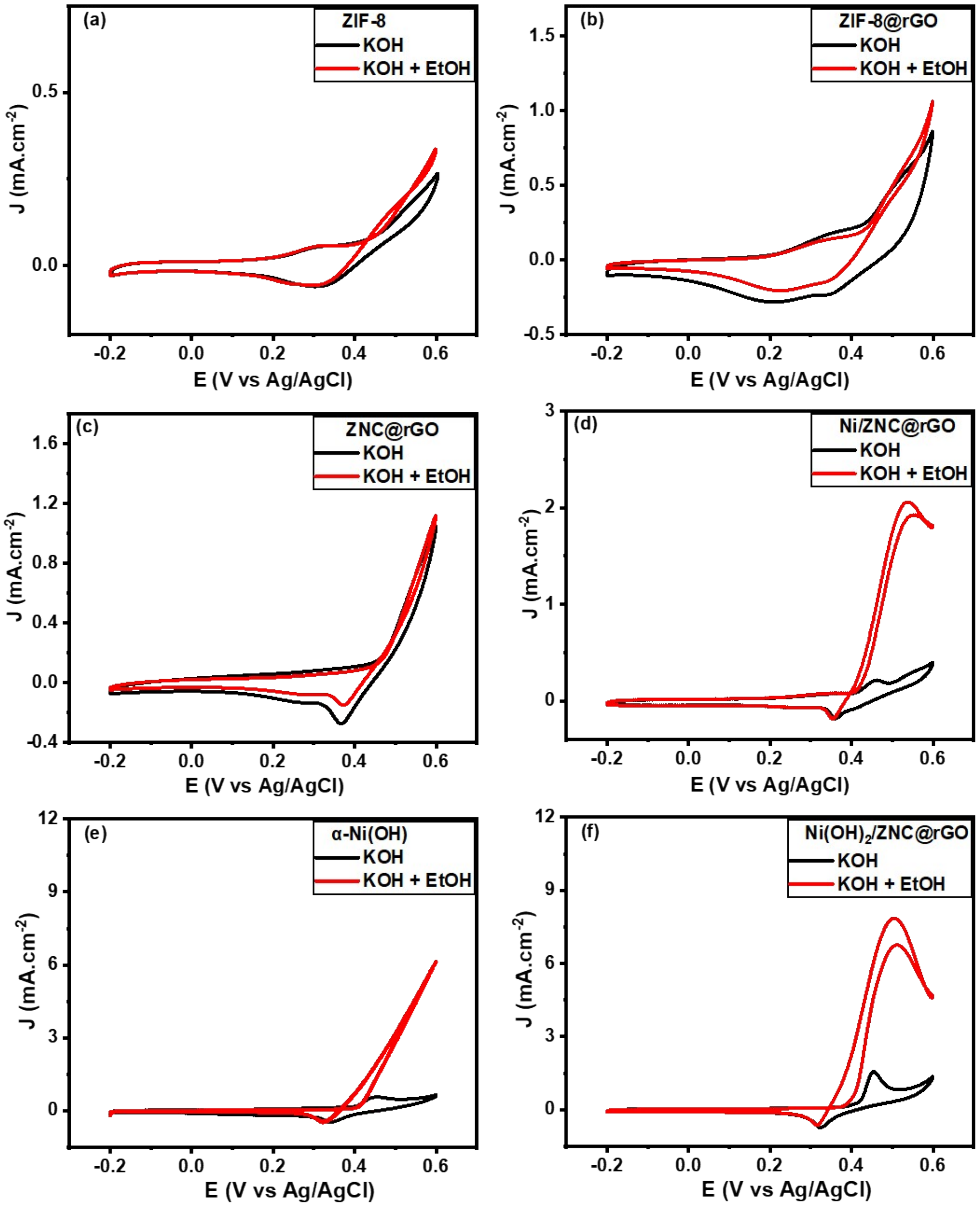


**Fig. S7:** CV curves of (a) ZIF-8, (b) ZIF-8@rGO, (c) ZNC@rGO, (d) Ni/ ZNC@rGO, (e) α-$Ni(OH)_2$ and (f) $Ni(OH)_2$/ ZNC@rGO in the absence and presence of 1M ethanol at a scan rate of 50 mV $s^{-1}$.

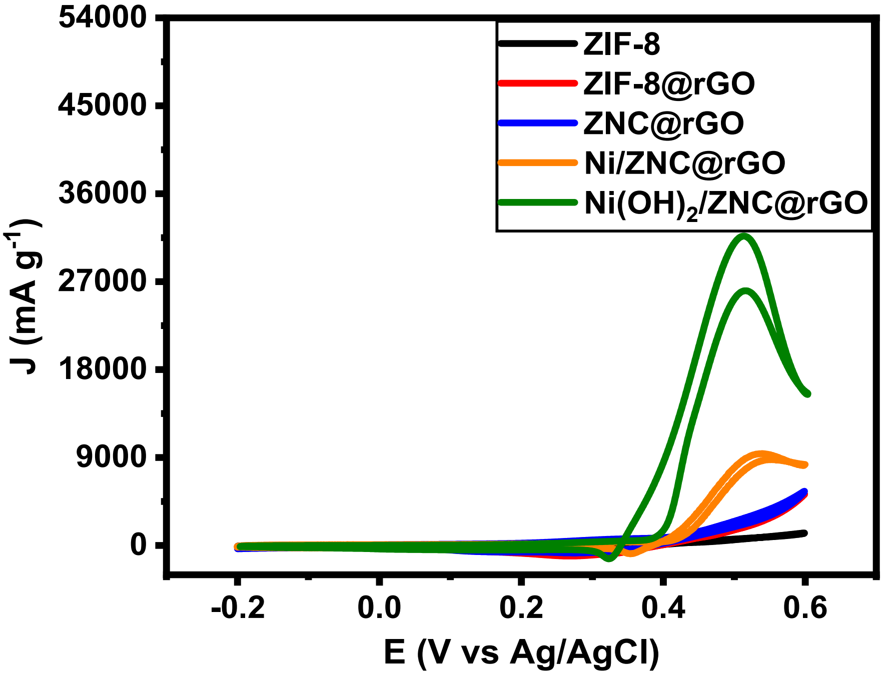


**Fig. S8:** CV curve of the as-prepared catalysts in 1M KOH + 1M Ethanol at a scan rate of 50 mV $s^{-1}$.

**Table 1: Comparison of the prepared catalyst for EOR efficiency with some modified electrodes vs Ag/AgCl.**

| Working electrode | $C_{EtOH}$ (M) | $E_{onset}$ (V) | $E_{pa}$ (V) | $I_{pa}$ (mA/cm$^2$) | Ref. |
|---|---|---|---|---|---|
| NiCo/ErN-GO/CCE | 0.1 | 0.38 | 0.81 | 64.23 | 1 |
| Ni(II)(Phen)$_2$/rGO-modified PGE | 1.0 | 0.4 | 0.53 | 0.81 | |
| NiPPy/GCE | 0.9 | --- | 0.55 | 8.50 | 2 |
| Ni/p(NMA)/MCPE | 0.4 | 0.46 | 0.66 | 1.80 | 3 |
| CPE@[Ni(cis-salcn)] | 0.5 | 0.42 | 0.55 | 2.60 | 4 |
| CM-ZnO | 2.0 | 0.40 | 0.58 | 35.0 | 5 |
| $Cu_2O$/PPy/CPE | 0.2 | 0.58 | 0.80 | 2.25 | 6 |
| 4-trifluoromethylphenyl ( TFMP)-Pt/C | 1.0 | -0.489 | ----- | 776 | 7 |
| NiO@PPC-600 | 1.0 | 1.1 (vs RHE) | 1.4 (vs RHE) | 231.8 | 8 |
| Ni,$Fe_2O_3$/Cu<br>Cu/Ni,$Fe_2O_3$ | 1.0 | 0.31<br>0.31 | 0.476<br>0.510 | 0.492<br>0.644 | 9 |
| Microbead-encapsulated ZnO particles derived from coffee waste | 2.0 | 0.4 | 0.5 | 35 | 5 |
| Ag/$Ag_2O$ | 1.5 | 0.43 | 0.5 | 69 | 10 |
| **Ni(OH)$_2$/ ZNC@rGO** | **1.0** | **0.34** | **0.51** | **8.30** | **This work** |

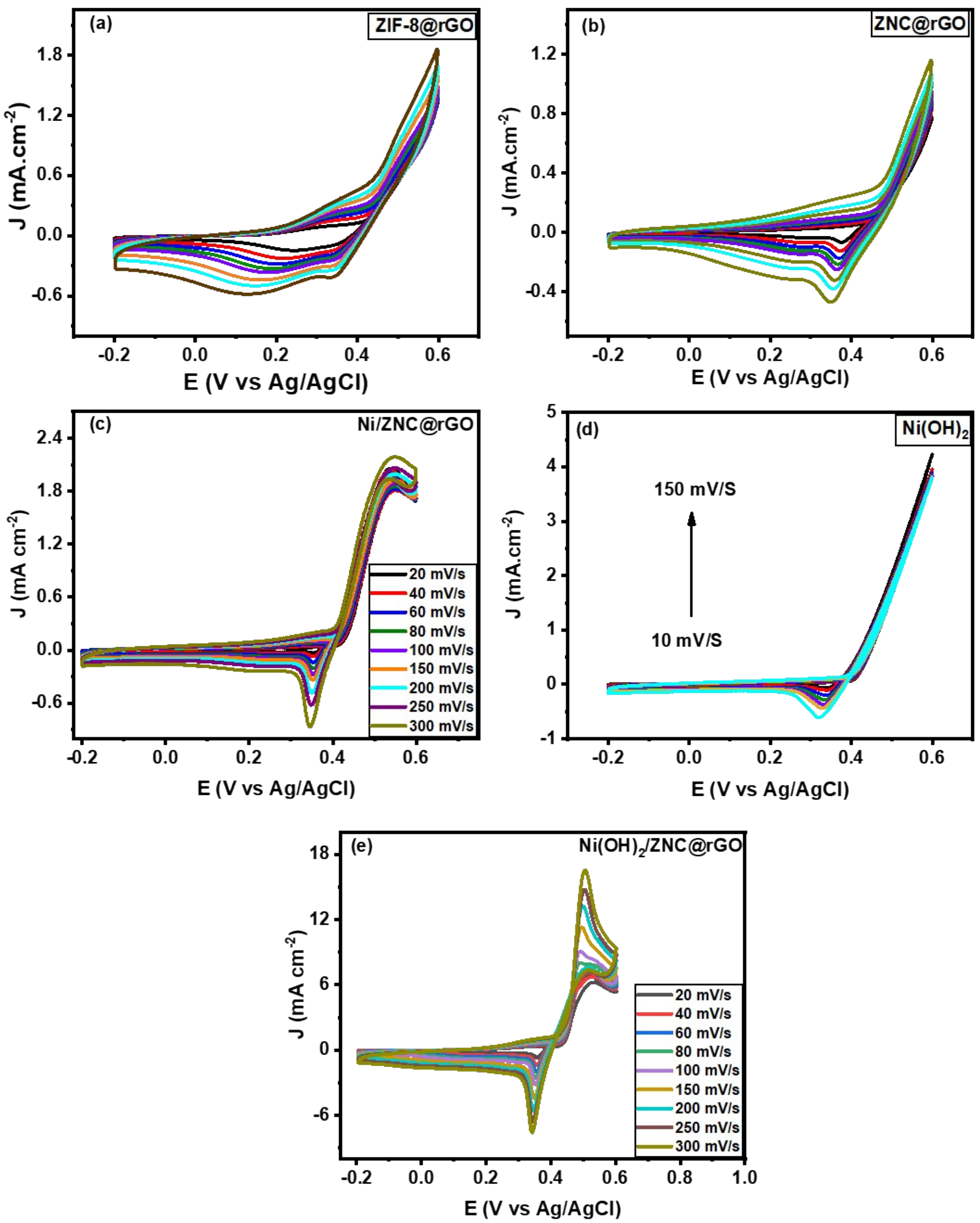


**Fig. S9:** CV curves recorded in 1M KOH in the potential range of -0.2- 0.6 V at different scan rates for (a) ZIF-8@rGO, (b) ZNC@rGO, (c) Ni/ ZNC@rGO, (d) α-$Ni(OH)_2$, and (e) $Ni(OH)_2$/ ZNC@rGO catalysts.

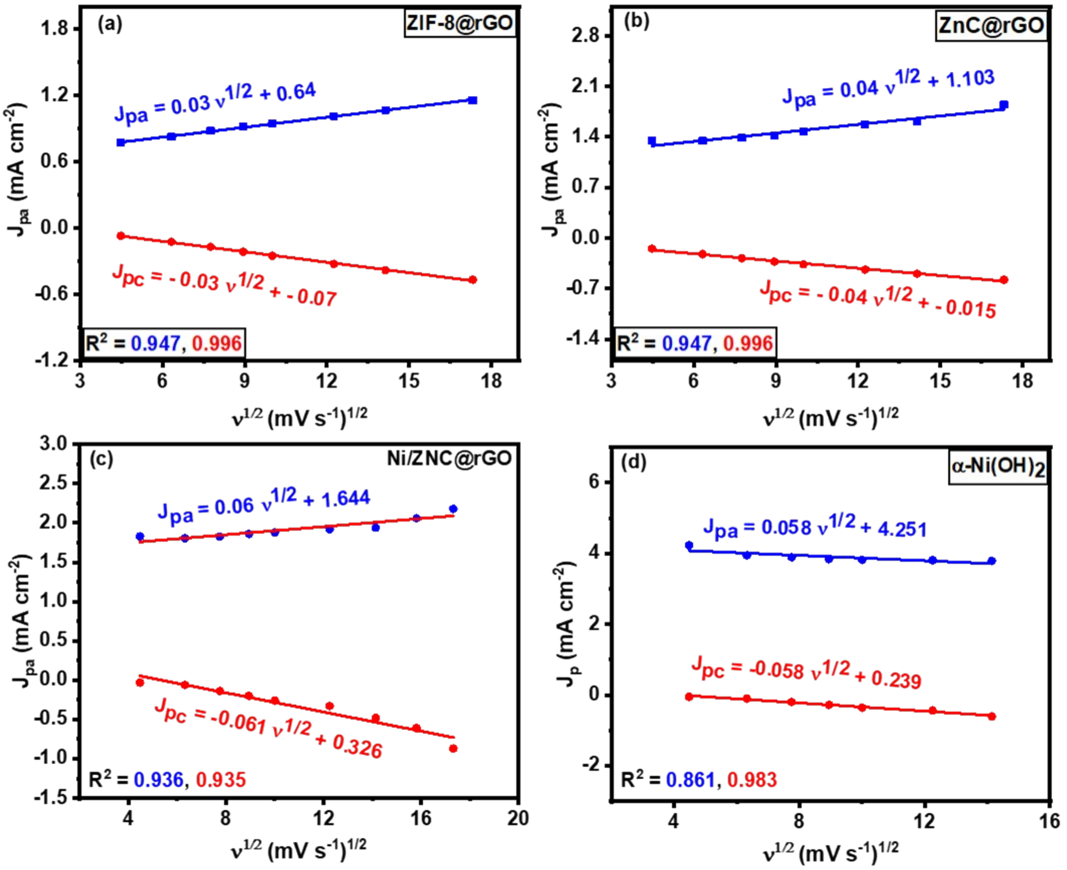


**Fig. S10:** Linear relationship of anodic ($I_{pa}$) and cathodic current ($I_{pc}$) densities of (a) ZIF-8@rGO, (b) ZNC@rGO, (c) Ni/ZNC@rGO, and (d) α-Ni(OH)$_2$ versus the square root of scan rates.

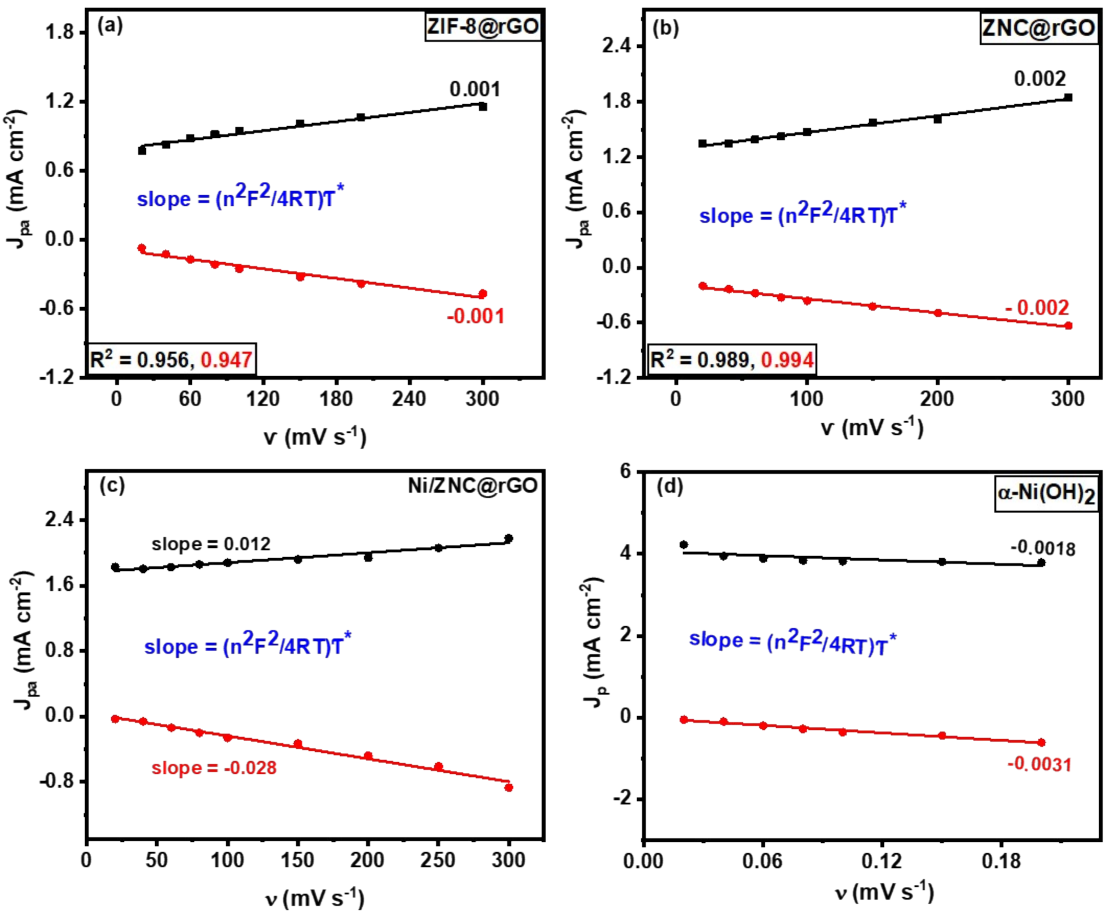


**Fig. S11:** Linear relationship of anodic ($I_{pa}$) and cathodic current ($I_{pc}$) densities of (a) ZIF-8@rGO, (b) ZNC@rGO, (c) Ni/ZNC@rGO, and (d) α-$Ni(OH)_2$ versus the scan rates.

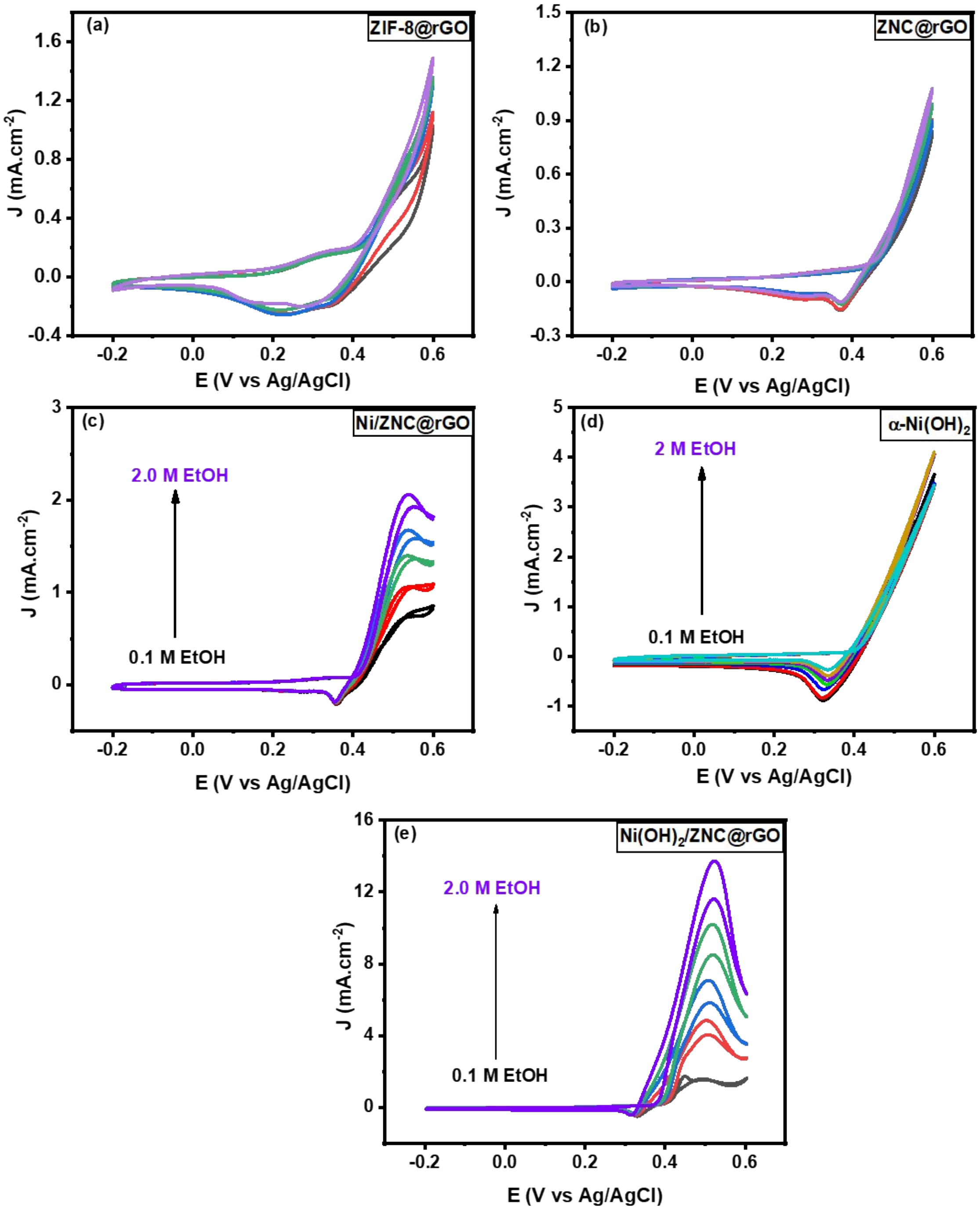


**Fig. S12:** CV of (a) ZIF-8@rGO, (b) ZNC@rGO, Ni/ ZNC@rGO, (d) α-Ni$(OH)_2$ and (e) Ni$(OH)_2$/ ZNC@rGO catalysts in 1M KOH with different concentrations of ethanol (0.1- 2.0 M).

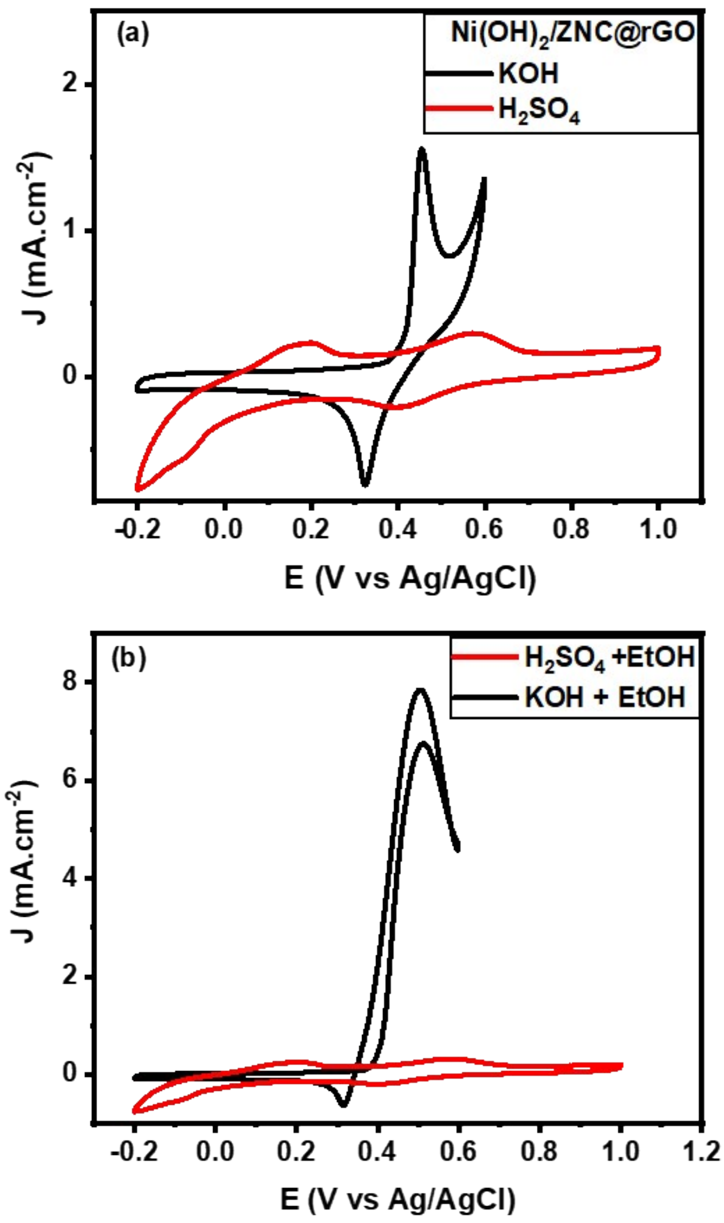


**Fig. S13**: CV of $Ni(OH)_2$/ ZNC@rGO in (a) 1M of alkaline and acidic electrolyte and (b) 1M of EtOH+ 1M of alkaline or acidic medium.

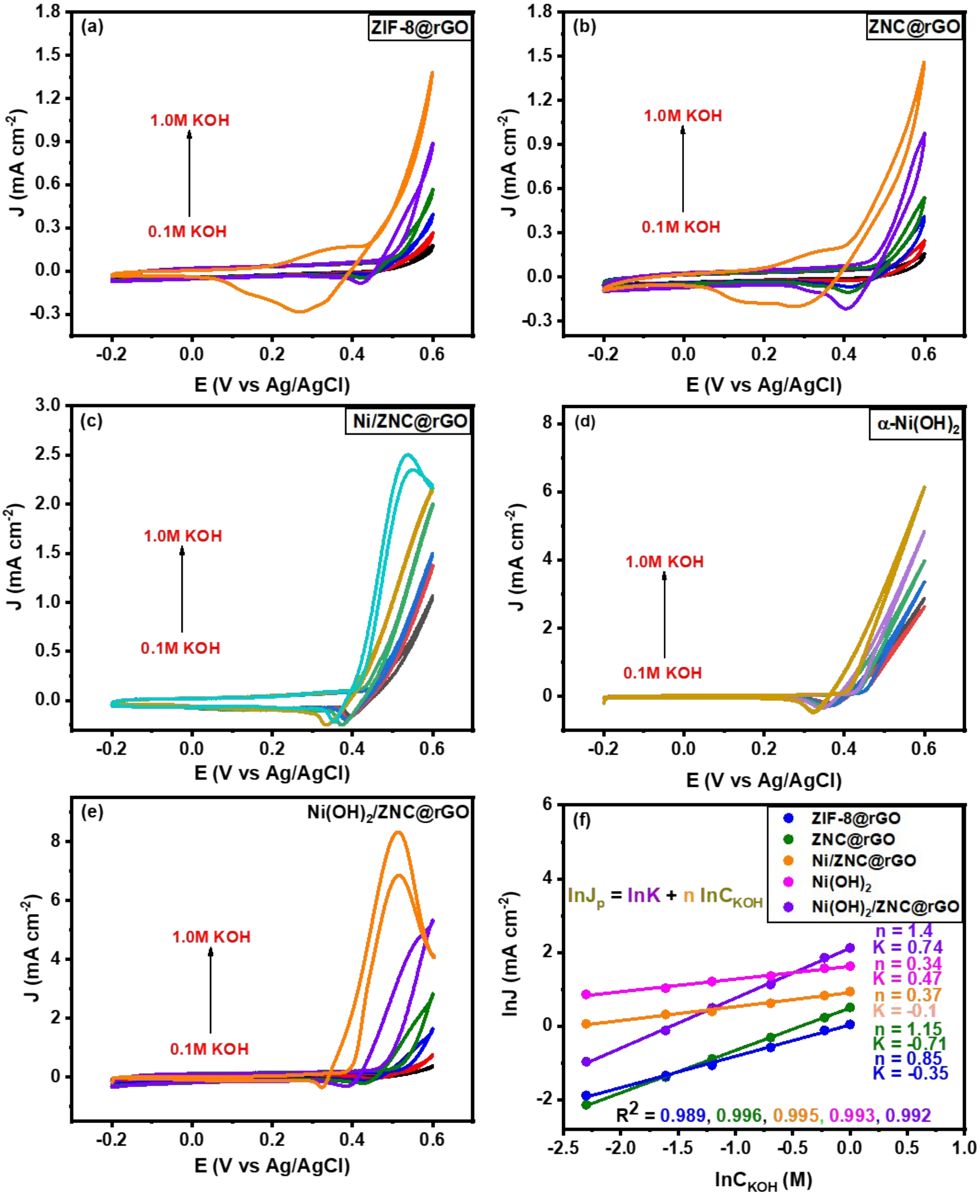


**Fig. S14:** CV of (a) ZIF-8@rGO, (b) ZNC@rGO, (c) Ni/ ZNC@rGO, (d) α-$Ni(OH)_2$ and (e) $Ni(OH)_2$/ ZNC@rGO catalysts in 1M EtOH with different concentrations of KOH electrolyte (0.1- 1.0 M) and (f) effect of concentration of the KOH electrolyte in electrocatalytic ethanol oxidation.

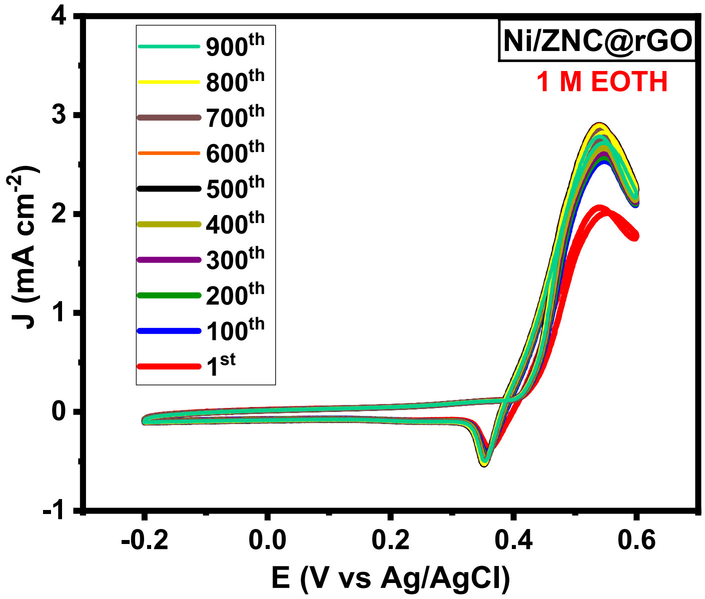


**Fig. S15:** CVs curves for 900 cycles from -0.2- 0.6 V at a scan rate of 10 mV $s^{-1}$ in 1M KOH+ 1M EtOH using Ni/ ZNC@rGO.

## References


1. K. Rahmani and B. Habibi, *RSC Adv*, 2019, **9**, 34050-34064.
2. A. Ehsani, M. G. Mahjani, M. Jafarian and A. Naeemy, *Electrochim Acta*, 2012, **71**, 128-133.
3. J.-B. Raoof, A. Omrani, R. Ojani and F. Monfared, *Journal of Macromolecular Science, Part A*, 2013.546-541 ,50 ,
4. G. V. Lima, L. dos Santos Mello, E. R. Dockal and M. Firmino de Oliveira, *Microchemical Journal*, 2022, **175**, 107209.
5. Z. K. Ghouri, K. Elsaid, A. Abdel-Wahab, A. Abdala and M. Z. Farhad, *J Mater Sci: Mater Electron*, 2020, **31**, 6530-6537.
6. A. El Attar, L. Oularbi, S. Chemchoub and M. El Rhazi, *Int J Hydrogen Energy*, 2020, **45**, 8887-8898.
7. C. Han, Y. Lyu, S. Wang, B. Liu, Y. Zhang, J. Lu and H. Du, *Carbon Energy*, 2023, **5**.
8. N. Gao, L. Gao, X. Zhang, Y. Zhang and T. Hu, *J Alloys Compd*, 20.169485 ,947 ,23
9. M. Ghalkhani, R. Abdullah Mirzaie, A. Banimostafa, E. Sohouli and E. Hashemi, *Int J Hydrogen Energy*, 2023, **48**, 21214-21223.
10. M. G. A. Saleh, S. A. El Wanees, I. D. Alruwaili, A. Akremi, A. M. Elbasiony and M. Brakat, *Int. J. Electrochem. Sci.*, 2023, **18**, 100372.
